# LAP2 Isoform Profile in Heart Ageing and in Cardiac Cell Proliferation and Differentiation: Input From CRISPR-Cas9-mediated LAP2a Knockdown in H9C2

**DOI:** 10.7150/ijms.114095

**Published:** 2026-01-21

**Authors:** Nathalie Vadrot, Maryline Moulin, Ana Ferreiro, Pascale Richard, Brigitte Buendia

**Affiliations:** 1 Université Paris Cité, CNRS, Unité de Biologie Fonctionnelle et Adaptative, F-75013 Paris, France.; 2 Université Paris Cité, CNRS, Institut Jacques Monod, F-75013 Paris, France.; 3 Université Paris Cité, CNRS, Epigenetics and Cell Fate, F-75013 Paris, France.; 4 APHP, Centre de référence des Maladies Neuromusculaires, Service de Neuromyologie, Institut de Myologie, CHU Pitié Salpêtrière- Charles Foix, Paris, France.; 5 UMR974 Inserm/Sorbonne Université, Centre de Recherche en Myologie, Paris, France.; 6 APHP, DMU BioGemH, HU Pitié Salpêtrière, Service de Biochimie Métabolique, Unité Fonctionnelle de Cardiogénétique et Myogénétique Moléculaire et Cellulaire, Paris, France.; 7 Sorbonne Université, INSERM, UMR_S 1166, IHU ICAN, Paris, France.

**Keywords:** LAP2, cardiomyopathy, heart, H9C2, cell proliferation, cell differentiation, ageing.

## Abstract

Haploinsufficiency of Lap2 alpha (LAP2a), a nuclear partner of Lamins A/C, has been associated with cardiac disease in rare cases, but LAP2a function remains largely unknown. To investigate the functional role of LAP2a in cardiomyocytes, we generated clones of embryonic myocardium-derived H9C2 cells in which LAP2a expression was specifically reduced through gene editing of the LAP2a gene *Tmpo* by CRISPR-Cas9.

Downregulation (+/-) and absence (-/-) of LAP2a expression led to a decreased proliferation capacity of cardiomyocytes *in vitro*. Upon differentiation, the expression of myocardial markers (alpha cardiac Actin 1/*Actc1*, cardiac Troponin T2/*Tnnt2*, Myosin-2/*Myh2* and Myosin-7/*Myh7*) was higher in LAP2a -/- cells compared to LAP2a +/- or LAP2a +/+ cells, with consistently higher expression of their upstream regulator *Mef2c* in LAP2a-devoid cells. These results suggest that LAP2a promotes cardiomyocyte proliferation and negatively modulates cardiomyocyte differentiation, through mechanisms including *Mef2c* regulation. Accordingly, normal protein expression of LAP2a was downregulated upon cardiomyocyte differentiation, contrary to LAP2b and a LAP2b-related shorter isoform. The latter tended to increase upon differentiation in all cells, most significantly in the LAP2a -/- clone.

In postnatal mouse hearts, LAP2a levels were higher in the right than in the left ventricle, and lowest in the septum. The LAP2a:LAP2b ratio was much lower in murine hearts than in H9C2 cells, and decreased significantly upon ageing, specifically in the left ventricle.

Finally, our data show that expression of the nuclear envelope proteins LEMD2 and Lamin A might be influenced by LAP2a upon cardiac differentiation.

Our results show that LAP2 expression is finely regulated upon cardiac differentiation *in vitro* and is dependent on age and heart compartment *in vivo.* They contribute to clarifying the potential impact of genetic LAP2a defects and their connection with heart disease, possibly including reduced cardiomyoblast proliferation, increased cardiomyocyte differentiation and altered nuclear envelope remodelling.

## Introduction

The nuclear envelope (NE) protein composition is not unique. It varies in function to the eukaryote species and also in function to the tissues and cellular environment 1-3. Such diversity in the NE protein composition contributes to optimizing the adaptability of the cell in terms of nuclear mechanics, genome organisation and gene expression 3-7. Accordingly, changes in the expression of several nuclear envelope proteins, as induced by genetic mutations, have been related to specific diseases 8. This includes Lamin A, which is encoded by the *LMNA* gene and whose genetic defects can affect either adipose or muscle tissues, or trigger premature ageing syndromes 9*.* While *LMNA* is a major gene associated with dilated cardiomyopathies*, TMPO,* which encodes the Lamin A partner LAP2alpha (LAP2a), has also recently been associated with cardiac diseases in male patients 10. However, the number of cases reported is low, and the role and expression pattern of LAP2a in the heart remains unclear.

LAP2a, a non-membranous nucleoplasmic protein, has multiple partners and is involved in different cellular functions such as cell proliferation and differentiation 11, genome organisation 12 and genome integrity 13. Interestingly, although LAP2a is a ubiquitous protein, its functions are modulated by its expression level and the cell/tissue context. For instance, reduced expression of LAP2a (induced by siRNA) has been correlated to low cell proliferation in human dermal fibroblasts 14 and in some cancer cells 15 16 17, but to a higher proliferation rate in epidermal/erythroid progenitor cells 18. In young mice, knockout of LAP2a (LAP2a -/-) triggered an increase in the muscle stem cell pool 19.

We have previously observed that a genetic *TMPO* mutation that triggered LAP2a haploinsufficiency was associated with hypertrophic cardiomyopathy (HCM) in two unrelated male patients 10. Interestingly, human dermal fibroblasts available from one of these patients showed a reduced proliferation capacity 10. It is not possible to confirm whether these results can be extended to patient-derived heart cells because of the inaccessibility of such cells.

Here, our first aim was to investigate the expression and potential role of LAP2a in a cardiomyocyte cellular model. We made use of cardiomyoblasts derived from an embryonic rat (H9C2) because they can expand when cultured in proliferation medium and present characteristics of early differentiation when transferred for 7 days into a specific medium that contains retinoic acid. Studying H9C2 cells makes it possible to assess the initial switch of cardiomyocytes from proliferation to differentiation, mostly related to heart embryogenesis. Indeed, the postnatal regeneration capacity of the heart is very limited and it further decreases with age 20,21.

We generated H9C2 clones with specifically reduced LAP2a expression after *Tmpo/*LAP2a gene editing by CRISPR-Cas9-mediated knockout (KO). We selected the viable clones and were thus able to analyse three heterozygous clones (LAP2a +/-) and one homozygous clone (LAP2a -/-). We observed a defect in cell proliferation for the LAP2a +/- and -/- clones compared to the LAP2a +/+ clones. In parallel, we followed the differentiation capacity of the clones by expression analysis of the cardiac markers alpha1 cardiac actin, cardiac troponin T2 and Myh7 2,22. The LAP2a +/- clones expressed these three early cardiac proteins similarly to WT clones. In comparison, the LAP2a -/- clone showed a higher expression of these cardiac markers, in accordance with the higher expression of the transcripts encoding the key cardiac transcription factors GATA4 and MEF2c together with the transcriptional coactivator p300 23-26. These data suggest that high LAP2a protein expression is not necessary to switch from proliferation to differentiation, but it does regulate their respective balance, at least *in vitro*, in favour of proliferation. If the role of LAP2a is more related to active cell proliferation, then one would expect its progressive downregulation in the heart upon ageing as cardiac cell renewal becomes more and more limited. To test this hypothesis, we evaluated the relative expression of LAP2a in WT mouse hearts at two ages, young adult (9 weeks) vs older adult (10 months). Indeed, the LAP2a level decreased from young to middle age. This decrease was drastic in the left ventricle, the sub-compartment of the heart that is preferentially affected in cardiomyopathy (HCM) related to LAP2a haploinsufficiency 10.

## Materials and Methods

### Proliferation and differentiation of H9C2 cardiomyoblasts

H9C2 cells (H9c2^2^-^1^ ATCC CRL-1446; LGC France) were initially derived from 13-day-old embryonic rat hearts, which mostly included ventricular tissue 27. At this time of development, cardiomyocytes still have a high capacity to proliferate 28,29. H9C2 cardiomyoblasts were grown in proliferation medium (DMEM medium containing high glucose (D6429, Merck/Sigma-Aldrich, France) and 10% FBS (Eurobio Scientific, France), in the presence of antibiotics (penicillin, streptomycin (15140122, ThermoFisher Scientific)). To promote cardiomyocyte differentiation, cells were seeded at 44,500 cells per well in 6-well plates. 24h later (Day 0), cells were incubated with differentiation medium (DMEM medium containing high glucose, 1% FBS, and 1 micromol Retinoic acid (sc-200898, Clinisciences, France) stock solution prepared at 10 mM in DMSO (D2650, Merck/Sigma-Aldrich, France). The differentiation medium was changed every day for 7 days. In parallel, cells were seeded at 6,400 cells per well in 6-well plates and grown in proliferation medium for 7 days.

### Generation of CRISPR-Cas9 KO LAP2alpha clones with H9C2 cells

**Figure 1:**
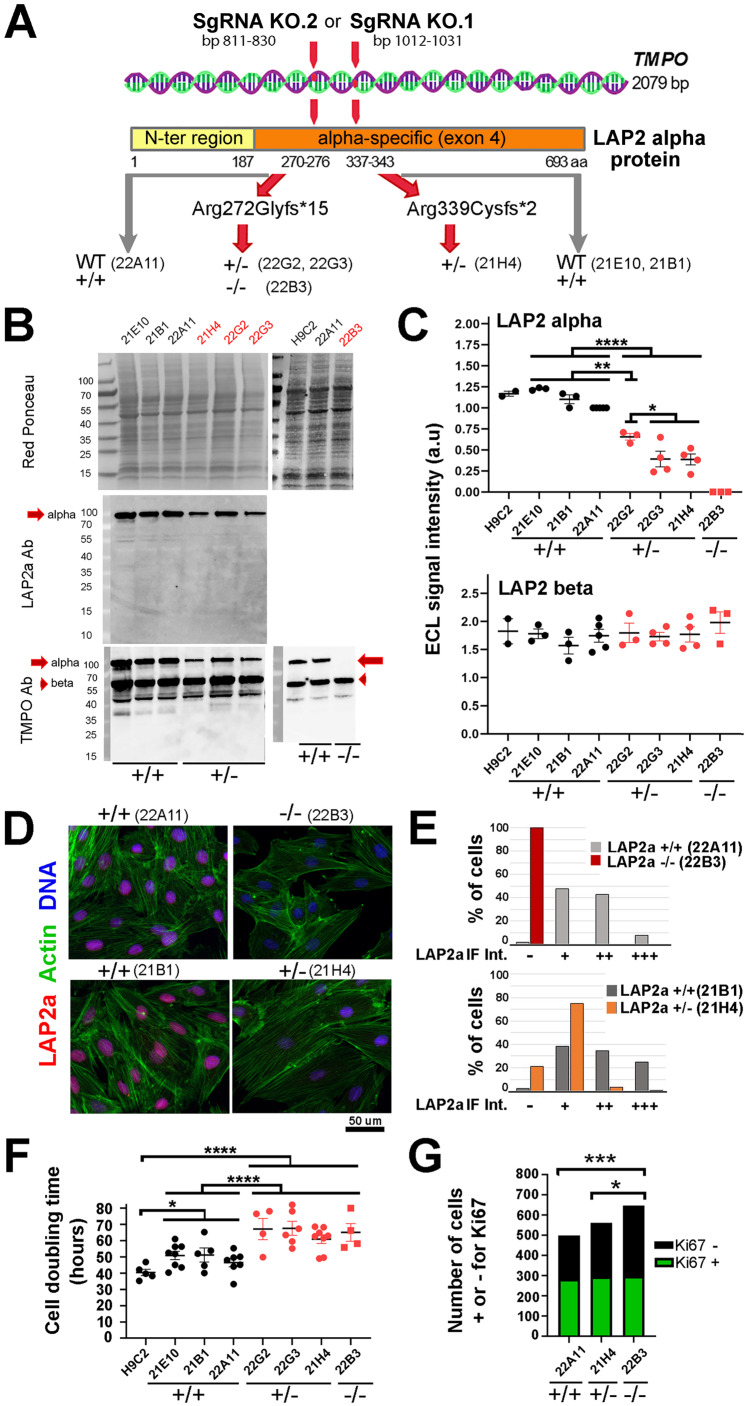
** Characterisation of H9C2 proliferating clones whose genome was modified within the *Tmpo* gene sequence by CRISPR-Cas9 to reduce or extinguish LAP2a protein expression.** (**A**) Schema of the *Tmpo* gene and LAP2a protein domains. The following are indicated: the number of base pairs targeted by one of the SgRNAs within the *Tmpo* gene and the amino acid position; the mutations induced by our SgRNA (at protein level) for the four H9C2 clones selected as effectively edited to trigger a premature stop codon; the names of the WT +/+ (unedited CRISPR clones) and of the LAP2a +/- and LAP2a -/- clones considered throughout this study. (**B**) Western blots are shown for whole protein extracts of naive H9C2 and CRISPR clones (WT (21E10, 21B1, 22A11); LAP2a +/- (21H4, 22G2, 22G3) and LAP2a -/- (22B3)), as indicated. Proteins of interest were detected using anti LAP2a Ab (middle panel) and anti TMPO Ab (lower panel). Red Ponceau staining (upper panel) was used to normalize ECL signals. (**C**) The graphs show the ECL signal quantification of western blots (arbitrary units; a.u) after revelation with anti TMPO Ab as shown in 1B. The graphs present the individual values and means ± s.e.m. (N= 2 to 5 independent samples per clone). * p<0.05, ** p<0.01, **** p<0.0001 (Mann Whitney test). (**D**) Immunofluorescence of CRISPR clones (WT (22A11, 21B1), LAP2a -/- (22B3) and LAP2a +/- (21H4)), using a rabbit Ab to detect LAP2a (red), phalloidin to label cytoplasmic actin (green) and DAPI to label nuclear DNA (blue). Scale bar = 50 um. (**E**) The graphs represent the % of cells with either a negative (-) or relatively higher or lower (+, ++, +++) mean signal (a.u) as detected by immunofluorescence when using a rabbit anti LAP2a Ab, as shown in 1D. For the analysed experiment (N = 1), the total n (numbers of cell nuclei) were 184, 321, 174 and 216 for the clones 22B3, 22A11, 21H4 and 21B1, respectively. (**F**) The graph represents the cell doubling time (mean value ± s.e.m.) calculated for naive H9C2 cells, WT CRISPR clones, LAP2a +/- and LAP2a -/- CRISPR edited clones, as indicated. The individual values and means ± s.e.m are given. (N = 4 to 8 independent experiments per clone). * p<0.05; **** p<0.0001. (Mann Whitney test) (**G**) The graph represents the amount of cells that were either positively (green) or negatively (black) stained *in situ* by immunofluorescence for the proliferation marker Ki67. For the analysed experiment (N = 1), the total n (numbers of cells) were 499, 562 and 647 for the WT, LAP2a +/- and LAP2a -/- clones, respectively. * p<0.05. *** p<0.001 (Chi square test for a contingency table).

H9C2 cells were seeded in 24-well plates at ~25,000 cells per well. 24h later, when the cells were 40-60% confluent, they were transfected with a mix of pure Cas9 protein (A36497, Protein V2 TrueCut Cas9, Life Technologies SAS, ThermoFisher Scientific, France) and a TrueGuide chemically modified SgRNA (Life Technologies SAS, ThermoFisher Scientific, France), in order to edit the exon 4 of rat *Tmpo*/LAP2a. The Cas9 and SgRNAs were mixed at 7.5 pmol each /well / 50µl, using the Lipofectamine CRISPR Max Cas9 transfection reagent (CMAX00001, Life Technologies SAS, ThermoFisher Scientific, France) and following the manufacturer's instructions. Two different SgRNA were used to perform two parallel transfection experiments. We designed the SgRNA KO.1 sequence as U*G*A*GGCUCCUCAGCACGCGA and the SgRNA KO.2 sequence as G*G*A*AUAAAUAAGCUCCGUCC, which corresponded to bp 1031-1012 and bp 830-811 of the reverse DNA sequence of rat *Tmpo*/LAP2a, respectively. Two days after transfection, the cells were detached and reseeded into 96-well plates after limiting dilution (i.e. targeting 0.8 cells/well). Growth of single cells was initially promoted by adding a high concentration of FBS (20% instead of 10%) in the proliferation medium. Amplification of clones was pursued and *Tmpo*/LAP2 gene sequencing was verified. We finally obtained several unedited clones (WT for *Tmpo*/LAP2a) and four clones (3 heterozygous and 1 homozygous) harbouring a *Tmpo* mutation associated with a frameshift and a premature stop codon (See Fig. 1 and Supplementary Data S1).

### Preparation of heart sub-compartments from young and adult mice

Nine-week-old (young) and ten-month-old (adult) male C57Bl6J mice were purchased from Envigo, (France). They were euthanized by cervical dislocation, and their hearts were rapidly excised (n = 10 each for young and adult mice). The hearts were dissected into left ventricle, right ventricle and septum and then snap-frozen in liquid nitrogen for further biochemical determinations. All animal experimental procedures complied with directive 2010/63/EU of the European Parliament on the protection of animals used for scientific purposes.

### qRT-PCR

RNAs were purified from H9C2 cells using the Nucleospin RNA kit (ref 740955.50, Macherey-Nagel, Germany). Then, RNAs were transcribed into cDNAs using the First strand cDNA synthesis kit for RT-PCR (ref 04896866001, Roche, Switzerland). qRT-PCR was performed to amplify mRNAs for *Lap2a*, *Lap2b*, *Lap2g*, *Lmna, Lmnb1, Emd, Tmem201, Klhl31, Popdc2, actc1, Tnnt2, Myh7, Gata4, Mef2c* and* P300* using specific primers (Primer List in Table 1). Relative mRNA quantification was performed by using *Hmbs*, a reference gene validated with the NormFinder algorithm. To summarise the data, the clones have been grouped into 3 categories, WT LAP2a +/+ (clones 21B1, 22A11), LAP2a +/- (clones 22G2, 22G3 and 21H4) and LAP2a -/- (clone 22B3).

### Antibodies

We used the following primary antibodies, according to the manufacturer's instructions: mouse anti-Actin alpha 1 cardiac muscle antibody (Ab) (33-32R, Novus Biological, BioTechne France), mouse anti-Histone H3 Ab (1G1, sc-517576; Santa Cruz Biotechnology, Germany), rabbit anti-Ki67 Ab (NB500-170; Novus Biological, BioTechne France), mouse anti-Lamin A/C Ab (4C11; Cell Signaling Technology, USA); rabbit anti-TMPO/LAP2 Ab (14651-1-AP; Proteintech Europe, United Kingdom) which recognizes all *TMPO* isoforms (a,b,g); mouse anti-Myosin-2 Ab (MF20, 14-6503-80, Life Technologies Europe, United Kingdom), rabbit anti-LAP2 alpha Ab (IQ175; Immuquest, Europe, United Kingdom); rabbit anti-Lamin B1 Ab 10; rabbit anti-LEMD2 Ab (HPA017340, Merck Sigma, France), mouse anti-cardiac troponin T monoclonal Ab (13-11; MA512960, Life Technologies, France) and phalloidin (P2141-Sigma).

### Immunofluorescence methods

For immunofluorescence (IF), H9C2 cells were fixed with paraformaldehyde 3% (sc-281692, Santa Cruz Biotechnology, Germany) for 12 min at R.T, then processed as described 10. The primary antibodies used were: rabbit anti-LAP2alpha Ab (1/250), rabbit anti-Ki67 Ab (1/35) and phalloidin (1/100). Immunofluorescence was observed using the confocal microscope Zeiss LSM 700 at the Imaging Facility at the BFA institute. Immunofluorescence signal intensities (relative mean intensity / nucleus) were estimated on confocal acquisitions, using ImageJ tools.

### Cell protein extract preparation and protein analysis by western blot

Whole protein extracts of cells were prepared by suspending cells directly in Laemmli sample buffer. Protein extracts from mouse hearts were prepared after tissue disruption with a tissue lyzer (Precellys, Bertin Technologies, France), as described previously 10. Proteins were analysed by immunoblot as described previously 30. ImageJ (Version 1.4.3.67) was used to quantify ECL signals.

### Proliferation capacity

Cells were cultured in growth medium, seeded at day 0 at the same density per plate. The total number of cells was then estimated by observation at the microscope of cells detached by trypsinization 1, 2, 3 and/or 4 days after. The calculation was made as follows: doubling time (hours) = [n hours / number of generations] with the number of generations = log2 (cell number fold-increase in n hours). In parallel, cells were processed for immunofluorescence with the rabbit anti-Ki67 Ab, and the proportion of positive vs negative cells was estimated with ImageJ on image acquisitions obtained using the confocal microscope (obj 10X).

## Results

### LAP2a modulates cardiomyoblast proliferation

*Tmpo* encodes several LAP2 isoforms that share their 3 first exons, whereas exon 4 is specific to LAP2alpha and exons 5 to 10 encode LAP2beta-like isoforms. We designed our SgRNAs in order to modify *Tmpo* in the exon 4. After growing the clones initially selected by limiting dilution (see Material and Methods), we performed *Tmpo* sequencing. We obtained 4 edited clones, 3 heterozygous and 1 homozygous, with potential effective knockout of LAP2a (Fig. 1A). The other clones were unedited and considered as WT controls (Fig. 1A) (Supplementary Data S1). With the SgRNA KO.1, we obtained one LAP2 +/- clone (21H4) harbouring a heterozygous mutation associated with a frameshift leading to a premature stop codon (PTC). The mutation (deletion of 2 bp) was expected to produce either no protein or a truncated protein (Arg339Cysfs*2) of ~37 kDa, shorter than the WT protein (693 a.a, ~75 kDa). With the SgRNA KO.2, we obtained three heterozygous clones harbouring a major mutation associated with a frameshift also leading to a PTC. This mutation (deletion of 1 bp) was expected to produce either no protein or a truncated protein (Arg272Glyfs*15) of ~31 kDa, much shorter than the WT protein (~75 kDa). One of the clones harboured the latter mutation in the homozygous state (LAP2a -/-; clone 22B3) (Supplementary Data S1). The sequencing of the two other clones (LAP2a +/-; clones 22G2, 22G3) revealed a WT allele and additional minor mutations expected to trigger frameshifts and premature stop codons (Supplementary Data S1). The diversity of *Tmpo*/LAP2a mutation sites suggests that the clones 22G2 and 22G3 might not have originated from one single cell, but possibly from two or more cells, despite the use of the limiting dilution to select the cells after transfection with the SgRNAs. Nevertheless, we considered these two populations as LAP2a +/- clones for the rest of our study.

Protein analysis by immunoblotting estimated a 2.2 fold mean reduction in the expression of LAP2a in the LAP2a +/- clones (ECL signal = 0.45 ± 0.06 for LAP2a +/- vs 1.00 for WT) and a total absence of LAP2a in the LAP2a -/- clone (Fig. 1B-C). Notably, in the LAP2a +/- cells, we only detected the full-length LAP2a but not the shorter forms expected to be between 31 and 37 kDa that might have resulted from the PTC mutations. This was confirmed when we used either the LAP2a Ab or the *TMPO*-LAP2 Ab that recognise a domain either specific to LAP2a (a.a 188-693) or common to all LAP2 isoforms (a.a 1-187), respectively. Thus, we obtained i) a significant downregulation and a complete absence of the wild-type form of LAP2a protein in the LAP2a +/- and LAP2a -/- clones, respectively and ii) the absence of any truncated LAP2a product in all of the clones studied here.

The other major *Tmpo* isoform, LAP2beta (LAP2b), was detected by western blot in similar amounts in WT and LAP2a +/- or -/- proliferating cells (Fig. 1C). This indicates that there is no “compensation” phenomenon between the two major isoforms at the protein level (LAP2 alpha and LAP2 beta), in the context of LAP2a downregulation in these rat cardiomyoblasts.

*In situ* staining of proliferating cells detected normally distributed LAP2a in the nucleoplasm of the WT and LAP2a +/- clones (Fig. 1D). However, we observed a decrease in the percentage of cells strongly positive for LAP2a in the LAP2a +/- cells vs the WT cells (comparison between 21H4 and 21B1 in Fig. 1D-E, lower panels). As expected, LAP2a could not be detected in LAP2a -/- cells (Fig. 1D-E; upper panels).

We then compared the proliferation capacity of our clones. Figure 1F shows that, in our cell culture conditions, the mean values of the doubling time for H9C2 and the WT LAP2a CRISPR clones were roughly similar (40.5 hours ± 1.9 vs 47.6 ± 1.6). In contrast, the LAP2a +/- and LAP2a -/- CRISPR clones presented a significantly longer cell doubling time (64.6 hours ± 2.3 and 65.1 hours ± 5.4; p=0.0001) (Fig. 1F). In parallel, we performed *in situ* staining of cells using an Ab directed against the proliferation marker Ki67 (Fig. 1G). The proportion of KI67-negative cells was significantly higher in the LAP2a -/- clone compared to the WT and LAP2a +/- clones (54.1% for LAP2a -/- vs 43.3% for WT (p=0.0003), and 47.5% for LAP2a +/- (p=0.02)) (Fig. 1G).

We conclude that a significant downregulation or absence of the LAP2a protein triggers a reduction in the H9C2 cardiomyoblast proliferation capacity.

### LAP2a is dispensable for cardiomyocyte early differentiation

**Figure 2:**
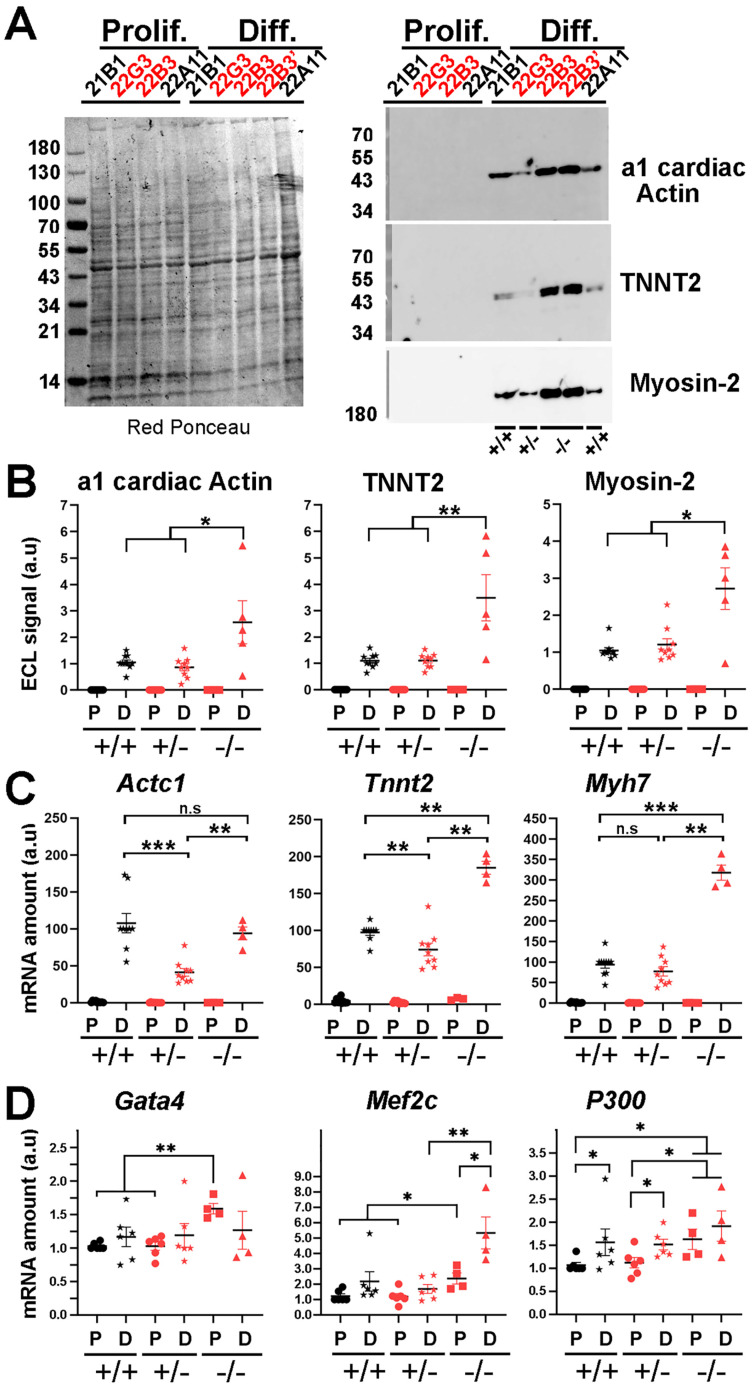
** Expression levels of cardiac markers and cardiac transcription factors in LAP2a CRISPR clones after 7 days in differentiation medium**. (**A**) Whole cell protein extracts were prepared from CRISPR clones (WT +/+, LAP2a +/- and LAP2a -/-) grown in proliferation (Prolif.) or incubated for 7 days in differentiation medium (Diff.) and analysed by western blot. The latter were revealed either with mouse anti Actin alpha 1 cardiac muscle Ab and successively with mouse anti cardiac troponin T monoclonal Ab, or with mouse anti Myosin-2 Ab. Red Ponceau staining prior to incubation with antibodies is also shown. (**B**) The graphs depict the relative protein amount (mean ± s.e.m normalized to Red Ponceau) for a1 cardiac Actin, cardiac troponin T2 (TNNT2) and Myosin-2 in cells grown in proliferation (P) or differentiation (D) medium. To summarise the data, the clones have been grouped into 3 categories: WT +/+ (21B1, 22A11), LAP2a +/- (22G2, 22G3 and 21H4) and LAP2a -/- (22B3). The graphs present the individual values and means ± s.e.m. (N = 4 or 5 independent differentiation experiments for each WT +/+ clone, N = 2 to 4 independent differentiation experiments for each LAP2a +/- clone; N = 4 or 5 independent differentiation experiments for the LAP2a -/- clone). * p<0.05, ** p<0.01 (Mann Whitney test). (**C**) The graphs depict relative mRNA levels normalized to *Hmbs* for *Actc1*, *Tnnt2* and *Myh7* in cells grown in proliferation (P) or differentiation (D) medium, as indicated. To summarise the data, the clones have been grouped into 3 categories: WT +/+ (21B1, 22A11), LAP2a +/- (22G2, 22G3 and 21H4) and LAP2a -/- (22B3). Each dot in the graphs represents the average value of a technical triplicate for RT-qPCR. The graphs also show the means ± s.e.m. (N = 4 or 5 independent differentiation experiments for each WT +/+ clone, N = 2 to 4 independent differentiation experiments for each LAP2a +/- clone; N = 4 or 5 independent differentiation experiments for the LAP2a -/- clone). ** p<0.01, *** p<0.001 (Mann Whitney test). (**D**) The graphs depict relative mRNA levels normalized to *Hmbs* for *Gata4*, *Mef2c* and *P300* in cells grown in proliferation (P) or differentiation (D) medium, as indicated. To summarise the data, the clones have been grouped into 3 categories: WT +/+ (21B1, 22A11), LAP2a +/- (22G2, 22G3 and 21H4) and LAP2a -/- (22B3). Each dot in the graphs represents the average value of a technical triplicate for RT-qPCR. The graphs also show the means ± s.e.m. (N = 3 independent differentiation experiments for each WT +/+ clone, N = 1 or 4 independent differentiation experiments for each LAP2a +/- clone; N = 4 independent differentiation experiments for the LAP2a -/- clone). * p<0.05, ** p<0.01, *** p<0.001 (Mann Whitney test).

To assess the putative role of LAP2a on early cardiac differentiation, our H9C2 CRISPR edited clones were incubated for 7 days in a differentiation medium containing retinoic acid but with a reduced concentration of serum (see Materials and Methods 22). We analysed the expression of alpha 1 cardiac actin (encoded by *Actc1*), TNNT2 (cardiac Troponin T2 encoded by *Tnnt2*), Myosin-2 (Type IIa myosin heavy chain encoded by *Myh2)* and Myosin-7 (Cardiac muscle myosin heavy chain 7 beta encoded by *Myh7)*. As expected, these differentiation markers were detected by western blot and by qRT-PCR only in cells transferred to the differentiation medium, but not in proliferating cardiomyoblasts (Fig. 2A-C). Protein levels of alpha 1 cardiac actin, TNNT2 and Myosin-2 were similar in the WT and LAP2a +/- clones and even higher in the LAP2a -/- CRISPR clone (~2.7 fold increase, ~3.3 fold increase and ~2.4 fold increase, respectively; Fig. 2B). The transcripts *Myh7* were similarly expressed in the WT clones and the LAP2a +/- clones, while the transcripts *Actc1* and *Tnnt2* were slightly less expressed in the LAP2a +/- clones than in the WT clones (Fig. 2C). The LAP2a -/- clone expressed these mRNAs at either comparable (*Actc1*) or even higher (*Tnnt2, Myh7*) levels than those observed in the WT clones (Fig. 2C).

From our data, we conclude that after 7 days of incubation into cardiac differentiation medium, i) the LAP2a +/- clones were as effective as the WT clones in inducing the expression of cardiac differentiation markers while ii) the LAP2a -/- clone (22B3) showed even a higher expression of the 3 differentiation markers considered here.

### LAP2a modulates *Gata4, Mef2c* and* P300* expression in cardiomyocytes

The fact that the LAP2a -/- cells were more effective than the LAP2a+/+ and LAP2a +/- cells in expressing the cardiac transcripts *Actc1*, *Tnnt2* and *Myh7* was somewhat intriguing. We thus tested the hypothesis that the expression of some key factors playing a role upstream for these 3 cardiac markers is specifically upregulated in the absence of LAP2a.

We focused our interest on* Mef2c*, *Gata4*, *P300* and *Hdac1* because they are known to regulate the expression of cardiac differentiation markers 23-26,31. qRT-PCR experiments revealed that the transcripts *Gata4*, *Mef2c* and *P300* were already expressed at day 0 in all clones (Fig. 2D). Moreover, at day 0, we observed a ~1.6 fold increase for both *Gata4* and *P300* and a ~2.0 fold increase for *Mef2c* in LAP2a -/- cells when compared to the WT or LAP2a +/- cells (Fig. 2D). At day 7 of differentiation, the expression of *Gata4* and *P300* was similar in all clones (Fig. 2D). Conversely, the expression of *Mef2c* was >2.5 times higher in the LAP2a -/- clone than in the other cells (Fig. 2D). As a comparison, the transcript *Hdac1* did not show significant changes in expression depending on the different LAP2a phenotypes, at either day 0 or day 7 of differentiation (data not shown).

Therefore, the capacity of the LAP2a -/- clone to express higher levels of *Gata4*, *Mef2c and P300* already at day 0 and to maintain higher levels of* Mef2c* at day 7 of differentiation is consistent with - and might contribute to explaining - the higher expression of cardiomyocyte differentiation markers (such as the proteins alpha 1 cardiac actin, TNNT2 and Myosin-2, and the transcripts *Tnnt2* and *Myh7*) observed in that clone.

### LAP2a expression is transcriptionally and post-transcriptionally downregulated upon cardiomyocyte differentiation

**Figure 3:**
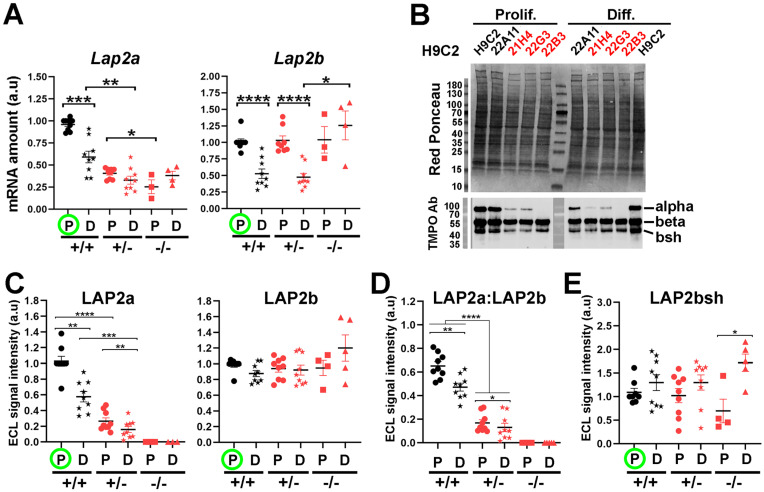
** Changes in LAP2a and LAP2b expression levels *in vitro* upon cardiac differentiation.** (**A**) mRNAs were prepared from CRISPR clones (WT +/+, LAP2a +/- and LAP2a -/-) grown in proliferation (P) or incubated for 7 days in differentiation medium (D). Their relative amount was evaluated by RT-qPCR using specific primers (see Materials and Methods). The graphs depict relative mRNA amount (normalized to *Hmbs*) for *Lap2a* and *Lap2b.* To summarise the data, the clones have been grouped into 3 categories: WT +/+ (21B1, 22A11), LAP2a +/- (22G2, 22G3 and 21H4) and LAP2a -/- (22B3). For each analysis, samples of LAP2a +/+ in proliferation (green circle) were used to arbitrarily define a reference value equal to 1.00. Each dot in the graphs represents the average value of a technical triplicate for RT-qPCR. The graphs also show the means ± s.e.m. (N = 4 or 5 independent differentiation experiments for each WT +/+ clone, N = 2 to 4 independent differentiation experiments for each LAP2a +/- clone; N = 3 or 4 independent differentiation experiments for the LAP2a -/- clone). * p<0.05; ** p<0.01; *** p<0.001; **** p<0.0001 (Mann Whitney test). (**B**) Whole cell protein extracts were prepared from CRISPR clones (WT +/+, LAP2a +/- and LAP2a -/-) grown in proliferation (Prolif., P) or incubated for 7 days in differentiation medium (Diff., D) and analysed by western blot. The latter were revealed with the rabbit anti TMPO Ab to detect LAP2a (alpha), LAP2b (beta) and a short LAP2b-like isoform (bsh). (**C-E**) The graphs depict the relative protein amount (i.e. mean ECL signal intensity (a.u) ± s.e.m normalized to Red ponceau) for LAP2a, LAP2 b, LAP2a:LAP2b, and LAP2bsh as indicated. To summarise the data, the clones have been grouped into 3 categories: WT +/+ (21B1, 22A11), LAP2a +/- (22G2, 22G3 and 21H4) and LAP2a -/- (22B3). For each analysis, samples of LAP2a +/+ in proliferation were used to arbitrarily define a reference value equal to 1.00 (green circle). The graphs present the individual values and means ± s.e.m. (N = 4 or 5 independent differentiation experiments for each WT +/+ clone, N = 2 to 4 independent differentiation experiments for each LAP2a +/- clone; N = 3 to 5 independent differentiation experiments for the LAP2a -/- clone). * p<0.05; ** p<0.01; *** p<0.001 (Mann Whitney test).

Our data showed that the LAP2a -/- clone was more effective in initiating cardiac differentiation compared to the LAP2a +/+ and LAP2a +/- clones. This led us to hypothesize that LAP2a negatively regulates cardiomyocyte differentiation, and thus a progressive and significant downregulation of LAP2a might be necessary for effective progress in this process. To evaluate this hypothesis, we analysed the relative expression level of LAP2a before and after differentiation in the WT and LAP2a +/- clones, at both the protein and transcript levels. In proliferating cells, the *Lap2a* mRNA levels were much lower in the LAP2a +/- and LAP2a -/- clones than in the WT clones, as expected (~2.3 fold and 3.8 fold reduction, respectively; Fig. 3A). Since the primers for RT-qPCR were designed upstream of the position of the KO mutations introduced by CRISPR-Cas9 (see Table 1 and Supplementary Data S2), they allowed amplification of both WT and mutant *Lap2a* mRNAs. Therefore, the lower amounts of *Lap2a* mRNA in the LAP2a +/- and -/- clones likely illustrates the instability of the mRNAs harbouring mutations in exon 4 of *Tmpo*. These mutant mRNAs are expected to be degraded by the non-sense mediated mRNA decay process, as we previously showed with a specific LAP2a_p.(Gly395Glufs*11) mutation identified in a patient 10. Additionally, cardiomyocyte differentiation triggered a strong reduction (~1.6 fold) of *Lap2a* mRNA in the WT clones, and a less marked decrease in the already deficient LAP2a +/- clones. Conversely, the LAP2a -/- clone showed a slight increase in *Lap2a* upon differentiation (Fig. 3A). However, the amount of *Lap2a* mRNA was still much lower in the LAP2a KO clone (~1.6 to 1.8 fold) than in the WT (Fig. 3A).

At the protein level, differentiation triggered a strong decrease of LAP2a both in the WT clones (~1.7 fold decrease) and in the LAP2a +/- clones (~1.6 fold decrease) (Fig. 3B-C). As expected, the LAP2a protein level remained systematically lower in the LAP2a +/- clones than in the WT LAP2a +/+ clones, both in proliferation and differentiation culture conditions (~3.8 and 3.6 fold reductions, respectively; Fig. 3C). In the LAP2a -/- clone, no mutant LAP2a protein was detected, both in the growth and differentiation medium (Fig. 3B-C), despite the accumulation of a residual level of mutant *Lap2a* mRNA (Fig. 3A). Therefore, the absence of mutant LAP2a protein likely results from an inhibition of the mutant *LAP2a* gene expression mostly at the transcriptional but also, to some extent, at the post-transcriptional level.

Altogether, these data show that a definite regulation of LAP2a expression occurs during the early steps of cardiac differentiation *in vitro*.

### The relative expression of the LAP2 isoforms (LAP2a, LAP2b and LAP2bsh) is modulated upon cardiomyocyte differentiation

In the WT and LAP2a +/- clones, we observed a marked reduction (~1.8 fold) of the *Lap2b* transcript level upon transfer into differentiation medium (Fig. 3A). However, in the same conditions, the protein expression of LAP2beta did not change significantly (Fig. 3B-C). The maintenance of high levels of LAP2b protein despite a significant reduction in *Lap2b* mRNA levels in differentiated cardiomyocytes suggests that a compensation mechanism must occur either at the translational and/or protein-degradation level 32. In contrast, in the LAP2a -/- clone, the *Lap2b* mRNA and LAP2b protein levels correlated well, remaining high before and after differentiation (Fig. 3A, C). Thus, the differentiation-dependent regulation of *Lap2b* mRNA seems to be dependent on LAP2a. Consistently, the LAP2a:LAP2b ratio decreased (~1.3 fold) in the WT and LAP2a +/- cardiomyocytes between proliferation and differentiation conditions (Fig. 3D). This decrease was mainly due to the decreased LAP2a expression (Fig. 3C).

Western blot using the rabbit anti-TMPO Ab also revealed a short LAP2b-like isoform, which we named LAP2bsh (LAP2 beta short). According to its apparent MW (~42kDa), it could be LAP2 delta and/or gamma. This shorter isoform was less represented than LAP2b (LAP2bsh:LAP2b = 0.33 ± 0.03 in cardiomyoblasts and 0.51 ± 0.05 in cardiomyocytes, p=0.019). LAP2bsh protein levels showed a tendency to increase upon cell differentiation in all clones, ranging from 1.3 times (WT and LAP2a +/- clones) to 2.4 times (LAP2a -/- clone; p<0.05) (Fig. 3E). Thus, the complete absence of LAP2a reinforced the increased expression of LAP2bsh protein associated with cardiac differentiation *in vitro*. Since we did not have the tools to precisely identify LAP2bsh, we did not try to further correlate the LAP2bsh protein level with the transcript level of one or other of the Lap2b-like isoforms.

### LAP2a expression in postnatal mouse hearts depends on the heart compartment and/or age

From our *in vitro* data, we hypothesised that progressive downregulation of LAP2a might further occur in the postnatal heart, when cardiomyocytes lose their proliferation capacity and become mature. To investigate this question, we analysed the expression level of LAP2a in the heart upon ageing, i.e. in young adult (Y; 9 weeks) and middle-aged adult (A; 10 months) mice.

**Figure 4:**
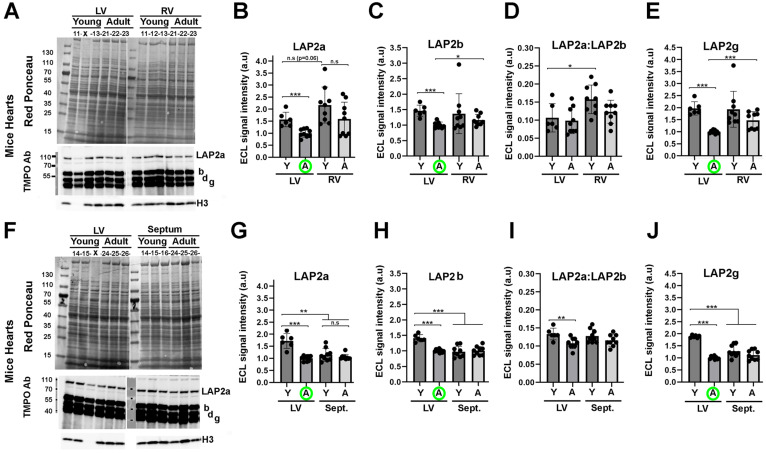
** Changes in LAP2a and LAP2b proteins expression levels *in vivo* upon heart ageing.** (**A, F**). Whole cell protein extracts were prepared from the left ventricle (LV), right ventricle (RV) and septum (Sept.), from male mice either young (9 weeks) or adults (10 months), as indicated and then analysed by western blot. The latter were revealed with the rabbit anti TMPO Ab to detect LAP2a and LAP2b and two shorter isoforms (LAP2d and LAP2g). The graph also shows the H3 detection that we used to verify that nuclei were correctly extracted in the samples, and if so to normalize the LAP2s ECL signals. Absence of H3 signal meant that nuclei were not optimally extracted (X); these samples were not considered for the further LAP2 quantification. (**B-E**) and (**G-J**) The graphs depict the relative protein amount (normalized to Histone 3) for LAP2a, LAP2b, LAP2a:LAP2b and LAP2g in the RV vs the LV or in the septum (Sept.) vs the LV, for young (Y) and Adult (A) mice, as indicated. For each analysis, the left ventricle (LV) samples from adult mice (A) were used to arbitrarily define a reference value equal to 1.00 (A; green circle). The graphs present the individual values and means ± s.e.m. (n = 6 mice for young LV, n = 9 mice for adult LV, n = 9 mice for young and adult RV, n = 9 mice for young and adult septum). * p<0.05; ** p<0.01; *** p<0.001 (Mann Whitney test).

Whole protein extracts were prepared from both the left ventricle (LV) and the right ventricle (RV), which show clear structural and functional differences 33. Additionally, structural/functional alterations of the LV or the RV are known to be associated with different heart diseases 33. We also prepared extracts from the interventricular septum, because it can be affected in human diseases and particularly in hypertrophic cardiomyopathy 34*.* Protein analysis by western blotting showed that LAP2a had a tendency to be more expressed in the RV than in the LV, both in young and adult mice (Fig. 4A-B). However, LAP2a was more expressed in the LV than in the septum, specifically in young mice (Fig. 4F-G). Upon ageing, a reduction of LAP2a was observed in both ventricles (Fig. 4B). This was particularly significant in the LV, where it reached a level as low as that observed in the septum whatever the age (Fig. 4G).

In conclusion, the data observed in mice are consistent with our *in vitro* findings, supporting a downregulation of LAP2a that correlates with the ageing of the heart *in vivo*, and occurs more significantly in the LV than in the RV.

### LAP2beta and LAP2beta-like short isoform protein expression decreases upon ageing in left ventricles of murine hearts

The change in LAP2b expression was not significant upon ageing in either the RV or the septum (Figs. 4A, 4C, 4F, 4H). In contrast, LAP2b expression was significantly reduced upon ageing in the LV (Figs. 4C, 4H). The LAP2a:LAP2b ratio was found to be either similar or reduced in the LV of young mice vs adult mice (Fig. 4D and 4I). These data illustrate a distinct LAP2a and LAP2b expression pattern in the different murine heart sub-compartments, which is also dependent on age specifically in the LV. Moreover, we noticed that the LAP2a:LAP2b ratio was 5 to 7 times lower (LAP2a:LAP2b roughly equal to 0.12) in the young and adult mouse heart sub-compartments than in the H9C2 +/+ cardiomyocytes (see Figs 3D vs 4D and 4I).

With the heart extracts, the rabbit anti-TMPO Ab for western blot revealed two LAP2 beta-like isoforms that, due to their molecular weights and the literature data, might correspond to LAP2 delta and LAP2 gamma (Figs. 4A, 4F). The expression level of these shorter isoforms changed upon ageing similarly to LAP2b, i.e. with a significant decrease upon ageing specifically in the LV (data for LAP2 gamma are shown in Figs. 4E, 4J; similar data for LAP2 delta are not shown).

Even though the potential roles of LAP2b-like short isoforms are not fully known, our results show that their expression is regulated both *in vitro* upon cardiac cell differentiation (Fig. 3) and *in vivo* upon ageing in the LV (Fig. 4).

### LAP2a modulates changes in the expression of nuclear envelope proteins which occur upon early cardiomyocyte differentiation

The NE structure results from complex interactions established between several proteins within the nuclear membranes and/or at its nuclear periphery. We investigated whether LAP2a downregulation might impact the expression of some of these neighbour or partner proteins. Among the LAP2a partners, we considered Lamins A/C and Emerin, whose primary genetic defects are associated with cardiac disease in cases of specific mutations 34-36. We also considered Lamin B, a LAP2b partner 37. In addition, we analysed the expression of TMEM201 (transmembrane protein 201), KLHL31 (Kelch Like Family Member 31), POPDC2 (Popeye Domain cAMP effector 2) and LEMD2 (LEM Domain nuclear envelope protein 2), four NE transmembrane proteins that are expressed in skeletal muscles and in the heart 2,38-40. Interestingly, a specific homozygous mutant of the LEMD2 and few heterozygous mutants of the POPDC2 genes have been shown to induce cardiac defects 40,41.

**Figure 5:**
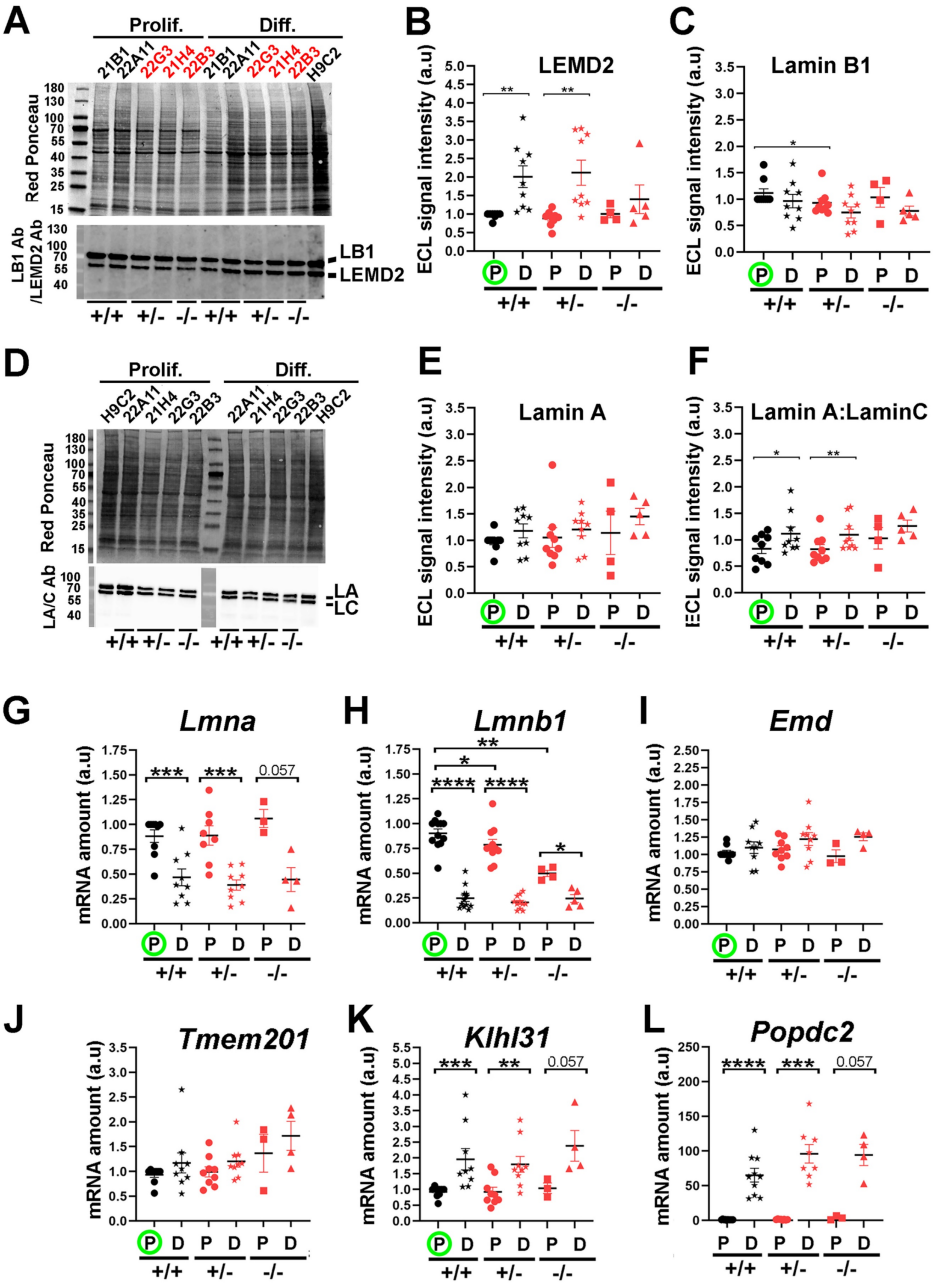
** Expression levels of NE markers after 7 days of cardiac differentiation**. (**A**) Whole cell protein extracts were prepared from CRISPR clones (WT +/+, LAP2a +/- and LAP2a -/-) grown in proliferation (Prolif.) or incubated for 7 days in differentiation medium (Diff.) and analysed by western blot. The latter were revealed with rabbit anti Lamin B1 Ab and successively with a rabbit anti LEMD2 Ab, as indicated. Red Ponceau staining prior to incubation with antibodies is also shown. (**B-C**) The graphs depict the relative protein amount (mean ± s.e.m normalized to Red Ponceau) for LEMD2 and Lamin B1 in cells incubated in proliferation (P) or differentiation (D) medium, as indicated. (**D**) Whole cell protein extracts were prepared from CRISPR clones (WT +/+, LAP2a +/- and LAP2a -/-) grown in proliferation (Prolif.) or incubated for 7 days in differentiation medium (Diff.) and analysed by western blot. The latter were revealed with rabbit anti Lamin A/C Ab. Red Ponceau staining prior to incubation with antibodies is also shown. (**E-F**) The graphs depict the relative protein amount (mean ± s.e.m normalized to Red Ponceau) for Lamin A and for the Lamin A:Lamin C ratio, as indicated. (**B-C**) and (**E-F**) To summarise the data, the clones have been grouped into 3 categories: WT +/+ (21B1, 22A11), LAP2a +/- (22G2, 22G3 and 21H4) and LAP2a -/- (22B3). The graphs present the individual values and means ± s.e.m. (N = 4 or 5 independent differentiation experiments for each WT +/+ clone, N = 2 to 4 independent differentiation experiments for each LAP2a +/- clone; N = 3 to 5 independent differentiation experiments for the LAP2a -/- clone). * p<0.05; ** p<0.01; *** p<0.001 (Mann Whitney test). (**G-L**) mRNAs were prepared from CRISPR clones (WT +/+, LAP2a +/- and LAP2a -/-) grown in proliferation (Prolif., P) or incubated for 7 days in differentiation medium (Diff., D). Their relative amount was evaluated by qRT-PCR using specific primers (see Materials and Methods). The graphs depict relative mRNA amount (normalized to *Hmbs*) for *Lmna, Lmnb1, Emd, Tmem201, Klhl31 and Popdc2*, as indicated. To summarise the data, the clones have been grouped into 3 categories: WT +/+ (21B1, 22A11), LAP2a +/- (22G2, 22G3 and 21H4) and LAP2a -/- (22B3). Each dot in the graphs represents the average value of a technical triplicate for RT-qPCR. The graphs also present the means ± s.e.m. (N = 4 to 7 independent differentiation experiments for each WT +/+ clone, N = 2 to 5 independent differentiation experiments for each LAP2a +/- clone; N = 3 to 5 independent differentiation experiments for the LAP2a -/- clone). * p<0.05; ** p<0.01; *** p<0.001; **** p<0.0001 (Mann Whitney test).

At the protein level, LEMD2 protein expression was significantly higher upon differentiation in the WT and LAP2a +/- clones (~2.0 to 2.4 fold increase) (Fig. 5A-B). In contrast, LEMD2 was still expressed at low levels in the LAP2a -/- clone upon differentiation (at levels comparable with those observed in proliferating LAP2a +/+ and LAP2a +/- cells) (Fig. 5A-B). Since all of the clones were able to initiate the cardiac differentiation process (Fig. 2), we conclude that the increase in LEMD2 protein levels might not be a key element at this early step of differentiation *in vitro*.

The expression levels of Lamin proteins did not change much upon differentiation in all of the clones. Nevertheless, we observed a decreasing trend for Lamin B1 (Fig. 5C) and an increasing trend for Lamin A (Fig. 5D-E), but these were not significant. We also noticed a significant increase (~1.33 fold) in the Lamin A:Lamin C ratio in both the WT and LAP2a +/- clones (Fig. 5F). At the mRNA level, both *Lmna* and *Lmnb1* decreased upon differentiation in all of the clones (Figs. 5G, 5H). The apparent discrepancy between the changes in mRNA and protein levels suggests an increase in the stability of proteins Lamin A and Lamin B1 upon differentiation.

Moreover, even in proliferating conditions, we noted that the LAP2a -/- clone had lower levels of *Lmnb1* mRNA (~1.15 fold decrease for the LAP2a +/- clones, p= 0.035; ~1.84 fold decrease for the LAP2a -/- clone, p= 0.001; Fig. 4B), but similar levels of Lamin B1 protein in comparison to the WT clones (Fig. 5C). This suggests that the stability of Lamin B1 protein might be higher in the LAP2a -/- clone than in the WT clones cultured in growth medium, possibly due to changes in post-translational modifications 42.

Notably, we found that the decrease in the transcription of genes encoding NE proteins such as *Lap2a*, *Lap2b*, *Lmna* and *Lmnb1* (Fig. 3A and Figs. 5G, 5H) was not a general rule during the early cardiac differentiation induced *in vitro*. Indeed, upon cardiac differentiation, all clones showed no significant change for *Emd* and *Tmem201* (Figs. 5I, 5J), but an increase for *Klhl31* (~1.9 to ~2.4 fold increase; Fig. 5K) and *Popdc2* (>50 fold increase; Fig. 5L). Intriguingly, expression of *Tmem201* and *Klhl31* tended to be higher in the LAP2a -/- cells compared to the LAP2a +/- or LAP2a +/+ cells at day 7 of differentiation, which might contribute to the cardiac differentiation process (see Discussion).

In conclusion, we show that the genes encoding the different NE components were not all regulated in the same way (increase, decrease or no change) upon cardiac differentiation of WT cells. Additionally, the increase of LEMD2 protein associated with differentiation was prevented in the LAP2a KO cells. Our results suggest that, upon early cardiomyocyte differentiation, LAP2a modulates the expression of genes encoding NE proteins, positively for *Lemd2* and negatively in the case of *Tmem201*, *Klhl31* and *Popdc2*.

## Discussion

### NE remodelling occurs upon *in vitro* cardiomyocyte differentiation

Remodelling of the NE is expected to trigger adaptations in terms of mechanics and/or genome regulation 2,39,43. Upon differentiation of WT cardiomyocytes *in vitro*, we observed changes in the expression levels of NE marker transcripts, including an increase in *Klhl31* and *Popdc2* but a decrease in *Tmpo/Lap2a*, *Tmpo/Lap2b*, *Lmna* and *Lmnb1*. At the protein level, we confirmed a decreased expression of LAP2a upon cardiac differentiation, in accordance with the *Lap2a* transcript level. In contrast, we found no changes in the protein expression of LAP2beta and Lamin B1 and only a slight increase in Lamin A. Our results differ from Kankeu et al's 2018 study 44*,* which noted a decrease of these markers upon differentiation. This apparent discrepancy might result from the different experimental approaches used to evaluate the relative amount of proteins (a global proteomic study in Kankeu et al. 2018 44, vs western blotting in our study) or from a slight variation in the kinetics of differentiation in the two studies.

The fact that the levels of Lamin proteins compared to the mRNA levels were higher than expected suggest that the stability of Lamin A and Lamin B1 proteins increases during cardiac differentiation. This is particularly relevant given that the half-lives of Lamins A/C and B1 have been reported to be particularly high in the heart (~several weeks (45).

We observed some differences in the LAP2a -/- clone vs the other cells for the expression of some NE markers upon cardiac differentiation (no LEMD2 increase, higher increase in *Tmem201, Klhl31* and* Popdc2* transcripts). We also noted that, in proliferation medium, the LAP2a +/- and LAP2a -/- clones expressed a decreased *Lmnb1* mRNA level but a similar Lamin B1 protein level compared to the WT clones. Our interpretation is that the stability of the Lamin B1 protein could be even higher in the case of LAP2a haploinsufficiency or knockout.

Therefore, our data illustrate changes in the expression of some nuclear markers indicative of NE remodelling, dependent on the cardiac cell differentiation state. They also show that these changes rely on transcriptional regulation and/or post-transcriptional modifications that might change the half-life of certain proteins 42. Finally, changes in the NE markers associated with LAP2a absence suggest an alteration of the NE remodelling that should normally occur upon cardiac differentiation. Whether these changes might alter the fate of cardiomyocytes either at late time points of differentiation (i.e. after day 7) or when subjected to specific environmental conditions (mechanical or oxidative stress...) remains to be studied.

### Fine regulation of LAP2 isoform expression occurs in the cardiac context

The *Tmpo* gene encodes several LAP2 isoforms by alternative mRNA splicing, whose regulation is tissue-dependent 46,47. Here, we show that the regulation of the *Tmp*o gene expression is dependent on the cardiac differentiation state of H9C2 cells, and involves both transcriptional and post-transcriptional regulation. As a consequence, cardiac differentiation is associated with a decrease in LAP2a, no change in LAP2b and an increase in LAP2b-like short protein steady state levels.

As expected, the LAP2a transcript level was strongly reduced in LAP2a +/- cells vs LAP2a +/+ cells. The fact that a residual expression of *Lap2a* occurred in the LAP2a -/- clone suggests a post-transcriptional regulation to prevent protein expression. Also, in the LAP2a -/- clone, both *Lap2a* and *Lap2b* slightly increased upon differentiation instead of decreasing as seen in both the WT and LAP2a +/- cells. Our interpretation is that the *Tmpo* gene transcription might be globally overactivated or that the stability of the *Tmpo* mRNAs might be higher in the LAP2a -/- differentiated cardiomyocytes than in the other cells.

Interestingly, the LAP2a:LAP2b ratio, which decreases by 1.4 fold upon cardiac differentiation *in vitro* in WT cells, is even lower in hearts from young and adult mice (a further 3.1 fold reduction). In the mouse heart, LAP2a is definitely a very minor isoform in terms of relative amounts as estimated in the LV, RV and septum. Instead, LAP2b and two additional LAP2b-like isoforms are abundant. This diversity in LAP2 isoforms is in accord with the transcript analysis on human foetal and adult hearts 48.

Surprisingly, we observed that the expression pattern of LAP2 proteins was quite distinct in the LV compared to the RV or the septum, and also varied in function to age. It is in the LV that modulation of all LAP2 isoform expression is the most sensitive to ageing, i.e. between young and middle-aged adults.

Therefore, it is not only LAP2a expression but also LAP2beta-like isoforms that appear to be finely regulated in the different sub-compartments of the heart, with an additional age-dependent regulation specifically in the LV. This suggests that there might be an adapted regulation of the alternative splicing of *Tmpo* in mouse hearts. Indeed, regulation of alternative splicing is a fundamental contributor to organ development, especially for the brain and the heart 49. Among the known splicing factors, there is hnRNPU which plays a key role in postnatal heart development and function 50*.* Moreover, deletion of hnRNPU, which is associated with a severe lethal DCM phenotype in mutant mice, revealed many misregulated events in cardiomyocytes, including a change in the global expression of *Tmpo* 51*.* Thus, it is tempting to hypothesise that hnRNPU might be an important splicing factor contributing to the fine regulation of *Tmpo* in mouse hearts.

### From the negative impact of LAP2a downregulation on cardiomyocyte proliferation to heart disease: hypotheses

The fact that LAP2a contributes to the regulation of the cell cycle is attributable to its capacity to bind Lamin A and pRb 11. We previously showed that the fibroblasts from a patient with HCM associated with LAP2a haploinsufficiency had a reduced capacity to proliferate *in vitro* 10. We show here a similar effect in the context of rat cardiomyocytes in which LAP2a knockout (partial or complete) was induced by CRISPR-Cas9 genome editing.

In parallel to these *in vitro* studies, the relevance of cell proliferation control in the heart is based on different observations. We know that cardiomyocyte proliferation is mostly restricted to the foetal and neonatal heart. However, in adult human hearts, low levels of cardiomyocyte renewal do still occur with an estimated annual rate of 1% at the age of 20, but 0.3% at the age of 75 21*.* Additionally, although the renewal of cardiomyocytes is part of the homeostatic regulation in the adult heart, it is also essential to repair/regenerate the heart after eventual cardiac injuries. Cardiomyocytes are the most abundant cell subpopulation (~50% of the cells) in both ventricles and in the septum 52. Recent studies have suggested that the renewal of cardiomyocytes in postnatal hearts does not rely on stem precursor cells. Instead, these authors proposed that it relies on a rare subpopulation of cardiomyocytes 53, that undergo a stage of dedifferentiation and re-entry into the cell cycle 54,55. Here, we show cardiomyocyte proliferation deregulation *in vitro* in the absence of LAP2a. Based on our data, we hypothesise that, *in vivo*, LAP2a promotes proliferation, and that its deficiency might contribute to defects in homeostatic regulation and/or in heart repair in response to certain stimuli.

Also, as proposed by Lazar *et al*. 21, a loss of the heart's ability to regenerate the cardiomyocyte population properly would require a compensation by an initial increase in the size of the pre-existing cardiomyocytes (hypertrophy) in order to maintain the contractility of the heart. The hypertrophic response is characterized by an over-expression of cardiac markers and is regulated by the upstream regulatory factors Gata4, Mef2c and P300 24,56-58*.* Interestingly, we found that some cardiac markers and upstream regulatory factors were more expressed in the LAP2a -/- cells than in the LAP2a +/- or LAP2a+/+ cells. Altogether, this suggests that the absence of LAP2a might trigger a sustained pathological hypertrophic response *in vivo,* leading to an increased risk of heart failure.

While our work shows that the absence of LAP2a has an impact on the expression of cardiac differentiation markers and upstream regulators in cardiomyocytes, the underlying mechanisms remain obscure. We speculate that they may involve a disturbed remodelling of the NE, with consequences on some signalling pathways, based on our observation that the expression of *Klhl31* and* Tmem201* and* Popdc2* was slightly increased in LAP2a -/- differentiated cardiomyocytes vs WT or LAP2a +/- cells. Assuming that these changes also occur at the protein level, this might contribute to altering certain signalling pathways known to play a role in cardiac cells. POPDC2, a member of the Popeye domain containing gene family, is a cAMP effector protein whose absence has been linked to cardiac defects 41,59. However, its potential role in regulating early cardiac markers such as *Actc1*, *Tnnt2* and *Myh7* has not been studied to our knowledge. In contrast, we do know that the proteins KLHL31 and TMEM201 regulate the MAPK and TGF beta signalling pathways, respectively. In particular, KLHL31 has been shown to alter the MAPK signalling pathway by repressing the expression and activity of JNK and its downstream target, c-Jun 60, while c-Jun had been shown to be a negative regulator of cardiac genes 61. Thus, one might hypothesize that the high expression of *Klhl31,* which we observed in the LAP2a -/- cells at day 7, might contribute to stimulating the expression of cardiac markers. On the other hand, TMEM201 has been shown to bind/phosphorylate/activate SMAD2, illustrating the role of TGF beta signalling in migrating breast cancer cells 62. In another study, SMAD2 phosphorylation/activation triggered by TGFbeta was shown to induce and stimulate the differentiation of human cardiomyocyte progenitor cells *in vitro* 63. Further investigations are needed to clarify whether the high expression of TMEM201 in our LAP2a -/- cardiomyocytes might favour the expression of cardiac markers through stimulation of the TGFbeta/SMAD2 signalling pathway.

Finally, the impact of LAP2a downregulation/absence on cardiac cells and the heart might rely on other known functions of LAP2a, such as its contribution to genome organisation/expression 12 and/or genome integrity 13. Moreover, it is important to keep in mind that cardiac diseases such as HCM present morphological, histological, and clinical phenotypes that are the consequences of complex interactions among a large number of determinants, ranging from the causal genetic mutation to environmental factors 64.

However, if we assume that the fine regulation of LAP2a that we observed in the different sub-compartments of mice hearts would also occur in human hearts, we can speculate on the consequences of LAP2a haploinsufficiency caused by a *TMPO*/LAP2a genetic mutation. Based on our data, we propose that LAP2a haploinsufficiency might reduce the regeneration of new cardiomyocytes but increase their capacity to express mature cardiomyocyte markers, contributing to heart hypertrophy characterized by the thickening of the LV wall and septum.

### Limits of the study

- For our *in vitro* studies, we generated H9C2 clones with a *Tmpo* gene that was edited (or not) by CRIPSR-Cas9. This cellular model allowed us to study the initial step of cardiac differentiation. However, to explore the impact of LAP2a downregulation on later steps of cardiac differentiation, i.e. cardiomyocytes with organised sarcomeres that are able to contract, it will be necessary to develop alternative cellular models.
- We were only able to select one clone whose CRISPR-Cas9 genome editing induced complete knockout of LAP2a. The selection of only one LAP2a -/- clone was possibly due to the fact that it is very difficult for these cells to survive and proliferate during the process of the clonal selection. Therefore, the conclusion that complete LAP2 knockout (LAP2a -/-) may stimulate the transcription of genes encoding LAP2b-like short isoforms and early cardiac differentiation markers requires further validation.
- Our *in vivo* data revealing certain specificities of the *Tmpo*/LAP2s pattern in function to the heart sub-compartment and age are based on analyses of mice. We do not yet know whether these data can be extrapolated to the human heart, since heart development/growth are not identical in mice and humans 65. Additionally, although the anatomical characteristics of human and murine hearts are very similar at the postnatal stage, a few differences have been highlighted in the morphology of the trabeculae in the right vs the left ventricle and the interventricular septum in the two species 65. Therefore, we cannot exclude differences in the NE composition, and in particular the *Tmpo*/LAP2 expression pattern, between human and mouse heart sub-compartments.

## Supplementary Material

Supplementary data 1.

Supplementary data 2.

## Figures and Tables

**Table 1:** List of primer sequences

Rat Primer Sequences (5'-3')		
***Gene***	**Forward**	**Reverse**
Hmbs	TCTAGATGGCTCAGATAGCATGCA	TGGACCATCTTCTTGCTGAACA
Lap2α	GAAGCTGTTGAAGCTGAGGGA	CTTGTGTTCTTTCTTCTTTCCTTCG
Lap2b	AGAACTCCAAGGAGAAGGGTGG	TTGATTGGTCTGCGGCAACT
Lmna	ATCACCATCGTGGCTCCCACT	GTAGGAGCGGGTGACTAGGTT
Lmnb1	GGAAGAATCAGAGGCGAGCA	CCTCCCATTGGTTGATCCTGT
Emd	CCTATTGTGGGCTCCACTCG	GGAGGCTGAATCCAAGT
Tmem201	CATCAAACACCAGAACCGCC	CGAGGAACTGAAACTCTGCCC
Klhl31	AGCAATTTCTGCAGGTACGA	CACGTAGCACTCCAGGGAA
Popdc2	ACATCGTCCTTTGGAACGTC	AACCCAAACTTGGCAAACAG
actc1	ATGATGCTCCCAGAGCTGTC	TGTCGTCCCAGTTGGTGATA
Tnnt2	GACAGGATCGAAAAGCGTCG	AGCCTTCCTCCTGTTCTCCT
Myh7	AAGAAGGAGCAGGACACCAG	TCTGGTAGGTGAGCTCCTT
GATA4	CAACTGCCAGACTACCACCAC	CCATGGAGCTTCATGTAGAGG
MEF2c	CCAAATCTCCTCCCCCTATG	CTCCCATCGTAGGAACTGCT
P300	AGATTCAGAGGGCAGCAGAGAC	GCCATAGGAGGTGGGTTCATAC
HDAC1	GCCAGTCATGTCCAAAGTAATGG	ATTAGGGATCTCTGTGTCCAGG

## Data Availability

All data are included in the manuscript or supplementary materials. Further inquiries can be directed to the corresponding author.

## References

[1] Chen IHB, Huber M, Guan T, Bubeck A, Gerace L (2006). Nuclear envelope transmembrane proteins (NETs) that are up-regulated during myogenesis. BMC Cell Biol.

[2] Wong X, Luperchio TR, Reddy KL (2014). NET gains and losses: the role of changing nuclear envelope proteomes in genome regulation. Curr Opin Cell Biol.

[3] Padilla-Mejia NE, Makarov AA, Barlow LD, Butterfield ER, Field MC (2021). Evolution and diversification of the nuclear envelope. Nucleus.

[4] Swift J, Ivanovska IL, Buxboim A, Harada T, Dingal PCDP, Pinter J (2013). Nuclear Lamin-A Scales with Tissue Stiffness and Enhances Matrix-Directed Differentiation. Science.

[5] Pawar S, Kutay U (2021). The Diverse Cellular Functions of Inner Nuclear Membrane Proteins. Cold Spring Harb Perspect Biol.

[6] Alisafaei F, Jokhun DS, Shivashankar GV, Shenoy VB (2019). Regulation of nuclear architecture, mechanics, and nucleocytoplasmic shuttling of epigenetic factors by cell geometric constraints. Proc Natl Acad Sci.

[7] Donnaloja F, Carnevali F, Jacchetti E, Raimondi MT (2020). Lamin A/C Mechanotransduction in Laminopathies. Cells.

[8] Dobrzynska A, Gonzalo S, Shanahan C, Askjaer P (2016). The nuclear lamina in health and disease. Nucleus.

[9] Malashicheva A, Perepelina K (2021). Diversity of Nuclear Lamin A/C Action as a Key to Tissue-Specific Regulation of Cellular Identity in Health and Disease. Front Cell Dev Biol.

[10] Vadrot N, Ader F, Moulin M, Merlant M, Chapon F, Gandjbakhch E (2023). Abnormal Cellular Phenotypes Induced by Three TMPO/LAP2 Variants Identified in Men with Cardiomyopathies. Cells.

[11] Dorner D, Vlcek S, Foeger N, Gajewski A, Makolm C, Gotzmann J (2006). Lamina-associated polypeptide 2alpha regulates cell cycle progression and differentiation via the retinoblastoma-E2F pathway. J Cell Biol.

[12] Zhang S, Schones DE, Malicet C, Rochman M, Zhou M, Foisner R (2013). High Mobility Group Protein N5 (HMGN5) and Lamina-associated Polypeptide 2α (LAP2α) Interact and Reciprocally Affect Their Genome-wide Chromatin Organization. J Biol Chem.

[13] Bao K, Zhang Q, Liu S, Song N, Guo Q, Liu L (2022). LAP2α preserves genome integrity through assisting RPA deposition on damaged chromatin. Genome Biol.

[14] Pekovic V, Harborth J, Broers JLV, Ramaekers FCS, van Engelen B, Lammens M (2007). Nucleoplasmic LAP2α-lamin A complexes are required to maintain a proliferative state in human fibroblasts. J Cell Biol.

[15] Zhang L, Wang G, Chen S, Ding J, Ju S, Cao H (2016). Depletion of thymopoietin inhibits proliferation and induces cell cycle arrest/apoptosis in glioblastoma cells. World J Surg Oncol.

[16] Li Z, Feng Y, Zhang Z, Cao X, Lu X (2020). TMPO-AS1 promotes cell proliferation of thyroid cancer via sponging miR-498 to modulate TMPO. Cancer Cell Int.

[17] Sun DP, Liew PL, Lin CC, Hung ST, Chen TC, Fang CL (2019). Clinicopathologic and Prognostic Significance of Thymopoietin-α Overexpression in Gastric Cancer. J Cancer.

[18] Naetar N, Korbei B, Kozlov S, Kerenyi MA, Dorner D, Kral R (2008). Loss of nucleoplasmic LAP2α-lamin A complexes causes erythroid and epidermal progenitor hyperproliferation. Nat Cell Biol.

[19] Gotic I, Schmidt WM, Biadasiewicz K, Leschnik M, Spilka R, Braun J (2010). Loss of LAP2 alpha delays satellite cell differentiation and affects postnatal fiber-type determination. Stem Cells.

[20] Senyo SE, Steinhauser ML, Pizzimenti CL, Yang VK, Cai L, Wang M (2013). Mammalian Heart Renewal by Preexisting Cardiomyocytes. Nature.

[21] Lázár E, Sadek HA, Bergmann O (2017). Cardiomyocyte renewal in the human heart: insights from the fall-out. Eur Heart J.

[22] Branco AF, Pereira SP, Gonzalez S, Gusev O, Rizvanov AA, Oliveira PJ (2015). Gene Expression Profiling of H9c2 Myoblast Differentiation towards a Cardiac-Like Phenotype. PloS One.

[23] Morin S (2000). GATA-dependent recruitment of MEF2 proteins to target promoters. EMBO J.

[24] Akazawa H, Komuro I (2003). Roles of Cardiac Transcription Factors in Cardiac Hypertrophy. Circ Res.

[25] Greco CM, Kunderfranco P, Rubino M, Larcher V, Carullo P, Anselmo A (2016). DNA hydroxymethylation controls cardiomyocyte gene expression in development and hypertrophy. Nat Commun.

[26] Muncie-Vasic JM, Sinha T, Clark AP, Brower EF, Saucerman JJ, Black BL (2025). MEF2C controls segment-specific gene regulatory networks that direct heart tube morphogenesis. Genes Dev.

[27] Kimes B, Brandt B (1976). Properties of a clonal muscle cell line from rat heart. Exp Cell Res.

[28] Barak Y, Hemberger M, Sucov HM (2019). Phases and Mechanisms of Embryonic Cardiomyocyte Proliferation and Ventricular Wall Morphogenesis. Pediatr Cardiol.

[29] Günthel M, Barnett P, Christoffels VM (2018). Development, Proliferation, and Growth of the Mammalian Heart. Mol Ther.

[30] Vadrot N, Duband-Goulet I, Cabet E, Attanda W, Barateau A, Vicart P (2015). The p.R482W substitution in A-type lamins deregulates SREBP1 activity in Dunnigan-type familial partial lipodystrophy. Hum Mol Genet.

[31] Lu D feng, Yao Y, Su Z zhuo, Zeng Z hua, Xing X wen, He Z yu (2014). Downregulation of HDAC1 Is Involved in the Cardiomyocyte Differentiation from Mesenchymal Stem Cells in a Myocardial Microenvironment. PLoS ONE.

[32] Vogel C, Marcotte EM (2012). Insights into the regulation of protein abundance from proteomic and transcriptomic analyses. Nat Rev Genet.

[33] Bernal-Ramirez J, Díaz-Vesga MC, Talamilla M, Méndez A, Quiroga C, Garza-Cervantes JA (2021). Exploring Functional Differences between the Right and Left Ventricles to Better Understand Right Ventricular Dysfunction. Oxid Med Cell Longev.

[34] Richard P, Fressart V, Charron P, Hainque B (2010). Génétique des cardiomyopathies héréditaires. Pathol Biol.

[35] Bione S, Maestrini E, Rivella S, Mancini M, Regis S, Romeo G (1994). Identification of a novel X-linked gene responsible for Emery-Dreifuss muscular dystrophy. Nat Genet.

[36] Karst ML, Herron KJ, Olson TM (2008). X-Linked Nonsyndromic Sinus Node Dysfunction and Atrial Fibrillation Caused by Emerin Mutation. J Cardiovasc Electrophysiol.

[37] Furukawa K, Fritze CE, Gerace L (1998). The Major Nuclear Envelope Targeting Domain of LAP2 Coincides with Its Lamin Binding Region but Is Distinct from Its Chromatin Interaction Domain. J Biol Chem.

[38] Wilkie GS, Korfali N, Swanson SK, Malik P, Srsen V, Batrakou DG (2011). Several Novel Nuclear Envelope Transmembrane Proteins Identified in Skeletal Muscle Have Cytoskeletal Associations. Mol Cell Proteomics.

[39] Korfali N, Wilkie GS, Swanson SK, Srsen V, De Las Heras J, Batrakou DG (2012). The nuclear envelope proteome differs notably between tissues. Nucleus.

[40] Abdelfatah N, Chen R, Duff HJ, Seifer CM, Buffo I, Huculak C (2019). Characterization of a Unique Form of Arrhythmic Cardiomyopathy Caused by Recessive Mutation in LEMD2. JACC Basic Transl Sci.

[41] Gruscheski L, Brand T (2021). The Role of POPDC Proteins in Cardiac Pacemaking and Conduction. J Cardiovasc Dev Dis.

[42] Murray-Nerger LA, Cristea IM (2021). Lamin post-translational modifications: emerging toggles of nuclear organization and function. Trends Biochem Sci.

[43] Ungricht R, Kutay U (2017). Mechanisms and functions of nuclear envelope remodelling. Nat Rev Mol Cell Biol.

[44] Kankeu C, Clarke K, Van Haver D, Gevaert K, Impens F, Dittrich A (2018). Quantitative proteomics and systems analysis of cultured H9C2 cardiomyoblasts during differentiation over time supports a 'function follows form' model of differentiation. Mol Omics.

[45] Buchwalter A (2023). Intermediate, but not average: The unusual lives of the nuclear lamin proteins. Curr Opin Cell Biol.

[46] Harris CA, Andryuk PJ, Cline S, Chan HK, Natarajan A, Siekierka JJ (1994). Three distinct human thymopoietins are derived from alternatively spliced mRNAs. Proc Natl Acad Sci.

[47] Harris CA, Andryuk PJ, Cline SW, Mathew S, Siekierka JJ, Goldstein G (1995). Structure and mapping of the human thymopoietin (TMPO) gene and relationship of human TMPO beta to rat lamin-associated polypeptide 2. Genomics.

[48] Taylor MRG, Slavov D, Gajewski A, Vlcek S, Ku L, Fain PR (2005). Thymopoietin (lamina-associated polypeptide 2) gene mutation associated with dilated cardiomyopathy. Hum Mutat.

[49] Mazin PV, Khaitovich P, Cardoso-Moreira M, Kaessmann H (2021). Alternative splicing during mammalian organ development. Nat Genet.

[50] Ye J, Beetz N, O'Keeffe S, Tapia JC, Macpherson L, Chen WV (2015). hnRNP U protein is required for normal pre-mRNA splicing and postnatal heart development and function. Proc Natl Acad Sci.

[51] Montañés-Agudo P, Pinto YM, Creemers EE (2023). Splicing factors in the heart: Uncovering shared and unique targets. J Mol Cell Cardiol.

[52] Litviňuková M, Talavera-López C, Maatz H, Reichart D, Worth CL, Lindberg EL (2020). Cells of the adult human heart. Nature.

[53] Kimura W, Xiao F, Canseco DC, Muralidhar S, Thet S, Zhang HM (2015). Hypoxia fate mapping identifies cycling cardiomyocytes in the adult heart. Nature.

[54] Payan SM, Hubert F, Rochais F (2020). Cardiomyocyte proliferation, a target for cardiac regeneration. Biochim Biophys Acta BBA - Mol Cell Res.

[55] Salama ABM, Gebreil A, Mohamed TMA, Abouleisa RRE (2021). Induced Cardiomyocyte Proliferation: A Promising Approach to Cure Heart Failure. Int J Mol Sci.

[56] Zhou L, Liu J, Xiang M, Olson P, Guzzetta A, Zhang K (2017). Gata4 potentiates second heart field proliferation and Hedgehog signaling for cardiac septation. Proc Natl Acad Sci.

[57] Shimizu S, Sunagawa Y, Hajika N, Yorimitsu N, Katanasaka Y, Funamoto M (2022). Multimerization of the GATA4 transcription factor regulates transcriptional activity and cardiomyocyte hypertrophic response. Int J Biol Sci.

[58] Kolodziejczyk SM, Wang L, Balazsi K, DeRepentigny Y, Kothary R, Megeney LA (1999). MEF2 is upregulated during cardiac hypertrophy and is required for normal post-natal growth of the myocardium. Curr Biol.

[59] Amunjela JN, Swan AH, Brand T (2019). The Role of the Popeye Domain Containing Gene Family in Organ Homeostasis. Cells.

[60] Yu W, Li Y, Zhou X, Deng Y, Wang Z, Yuan W (2008). A Novel Human BTB-kelch Protein KLHL31, Strongly Expressed in Muscle and Heart, Inhibits Transcriptional Activities of TRE and SRE. Mol Cells.

[61] McBride K, Robitaille L, Tremblay S, Argentin S, Nemer M (1993). *fos/jun* Repression of Cardiac-Specific Transcription in Quiescent and Growth-Stimulated Myocytes is Targeted at a Tissue-Specific *cis* element. Mol Cell Biol.

[62] Kong Y, Zhang Y, Wang H, Kan W, Guo H, Liu Y (2022). Inner nuclear membrane protein TMEM201 promotes breast cancer metastasis by positive regulating TGFβ signaling. Oncogene.

[63] Goumans MJ, De Boer TP, Smits AM, Van Laake LW, Van Vliet P, Metz CHG (2008). TGF-β1 induces efficient differentiation of human cardiomyocyte progenitor cells into functional cardiomyocytes *in vitro*. Stem Cell Res.

[64] Marian AJ, Braunwald E (2017). Hypertrophic Cardiomyopathy: Genetics, Pathogenesis, Clinical Manifestations, Diagnosis, and Therapy. Circ Res.

[65] Wessels A, Sedmera D (2003). Developmental anatomy of the heart: a tale of mice and man. Physiol Genomics.

