# Supplementary material for: LAP2 Isoform Profile in Heart Ageing and in Cardiac Cell Proliferation and Differentiation: Input From CRISPR-Cas9-mediated LAP2a Knockdown in H9C2
Source: Int J Med Sci. 2026 Jan 21;23(3):741–57. doi: 10.7150/ijms.114095 (PMC12964562; doi:10.7150/ijms.114095)

LAP2 isoform profile in heart ageing and in cardiac cell proliferation and differentiation: input from CRISPR-Cas9-mediated LAP2a knockdown in H9C2

Nathalie Vadrot[1,2], Maryline Moulin[1,3], Ana Ferreiro[1,4,5], Pascale Richard[6,7] and Brigitte Buendia[1,2]

Supplementary Data S1

Results of *TMPO* DNA sequencing

for CRISPR-Cas9 KO LAP2a clones (H9C2 cells)

Clone 22G2……LAP2a +/-

YOUR UPLOAD

LABEL	GUIDE SEQUENCE(S)	CONTROL FILE	EXPERIMENT FILE
**22G2 245R 200622_Premixed-TS03500300-copie**	GGAATAAATAAGCTCCGTCC	CTRL H9C2 PCR2 245R_Premixed-TS03500304-copie.ab1	22G2 245R 200622_Premixed-TS03500300-copie.ab1

Analysis of 22G2 245R 200622_Premixed-TS03500300-copie

Contributions | Indel Distribution | Traces

Status	Guide Target	PAM Sequence	Indel %	Model Fit (R^2)	Knockout-Score
Succeeded	GGAATAAATAAGCTCCGTCC	AGG	72	0.96	72

POWERED BY SYNTHEGO ICE

RELATIVE CONTRIBUTION OF EACH SEQUENCE (NORMALIZED)

	INDEL	CONTRIBUTION	SEQUENCE
	-1	48%	ACAGACGCAGAAGTTAGCCCCTGGA ¦ -GGAGCTTATTTATTCCTTCAGAGTCTAGCTATGATAGATGTGTAGAGAA
	-37	24%	ACAGACGC----------------- ¦ --------------------AGAGTCTAGCTATGATAGATGTGTAGAGAA
+	0	24%	ACAGACGCAGAAGTTAGCCCCTGGA ¦ CGGAGCTTATTTATTCCTTCAGAGTCTAGCTATGATAGATGTGTAGAGAA

POWERED BY SYNTHEGO ICE

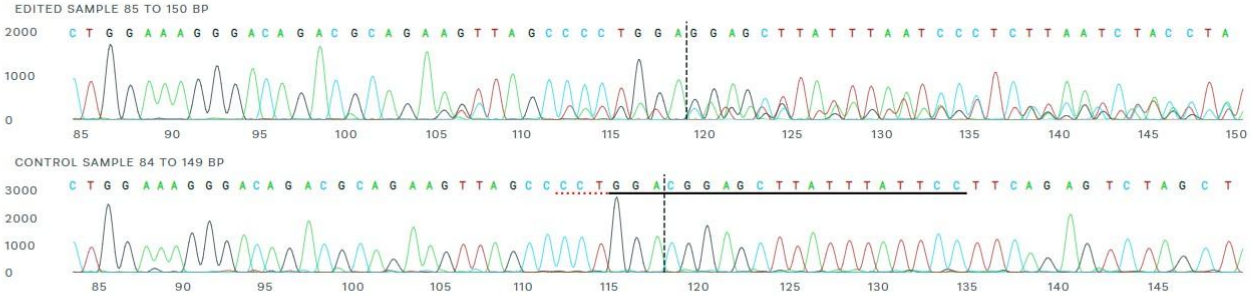

Clone 22G3……LAP2a +/-

YOUR UPLOAD

LABEL	GUIDE SEQUENCE(S)	CONTROL FILE	EXPERIMENT FILE
**22G3 245R 140223_Premixed-TS03935050**	GGAATAAATAAGCTCCGTCC	H9C2 22A11 245R 270622_Premixed-TS03543468.ab1	22G3 245R 140223_Premixed-TS03935050.ab1

Analysis of 22G3 245R 140223_Premixed-TS03935050

Contributions | **Indel Distribution** | **Traces**

Status	Guide Target	PAM Sequence	Indel %	Model Fit (R^2)	Knockout-Score
Succeeded	GGAATAAATAAGCTCCGTCC	AGG	71	0.97	71

POWERED BY SYNTHEGO ICE

RELATIVE CONTRIBUTION OF EACH SEQUENCE (NORMALIZED)

INDEL	CONTRIBUTION	SEQUENCE
-1	38%	ACTCTGAAGGAATAAATAAGCTCC- ¦ TCCAGGGGCTAACTTCTGCGTCTGTCCCTTTCCAGGCAAGGCTGTATCAG
+ 0	26%	ACTCTGAAGGAATAAATAAGCTCCG ¦ TCCAGGGGCTAACTTCTGCGTCTGTCCCTTTCCAGGCAAGGCTGTATCAG
+1	16%	ACTCTGAAGGAATAAATAAGCTCCG ¦ NTCCAGGGGCTAACTTCTGCGTCTGTCCCTTTCCAGGCAAGGCTGTATCA
-2	9%	ACTCTGAAGGAATAAATAAGCTCCG ¦ --CAGGGGCTAACTTCTGCGTCTGTCCCTTTCCAGGCAAGGCTGTATCAG
-2	7%	ACTCTGAAGGAATAAATAAGCTCC- ¦ -CCAGGGGCTAACTTCTGCGTCTGTCCCTTTCCAGGCAAGGCTGTATCAG
-2	1%	ACTCTGAAGGAATAAATAAGCTC-- ¦ TCCAGGGGCTAACTTCTGCGTCTGTCCCTTTCCAGGCAAGGCTGTATCAG

POWERED BY SYNTHEGO ICE

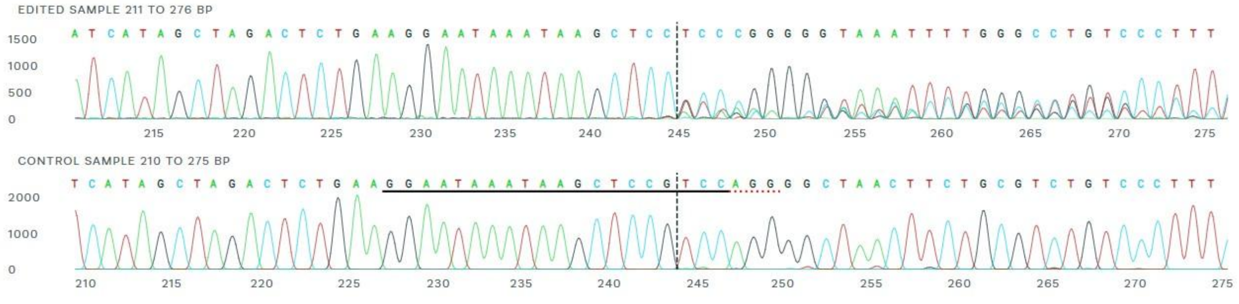

Clone 22B3….. LAP2a -/-

Analysis of 22B3 245FNP 070323_Premixed-TS03935074

Contributions | **Indel Distribution** | **Traces**

Status	Guide Target	PAM Sequence	Indel %	Model Fit (R^2)	Knockout-Score
Succeeded	GGAATAAATAAGCTCCGTCC	AGG	100	1	100

POWERED BY SYNTHEGO ICE

RELATIVE CONTRIBUTION OF EACH SEQUENCE (NORMALIZED)

INDEL	CONTRIBUTION	
-1	100%	C T G G A A A G G G A C A G A C G C A G A A G T T A G C C C C T G G A G G A G C T T A T T T A T T C C T T C A G A G T C T A G C T A

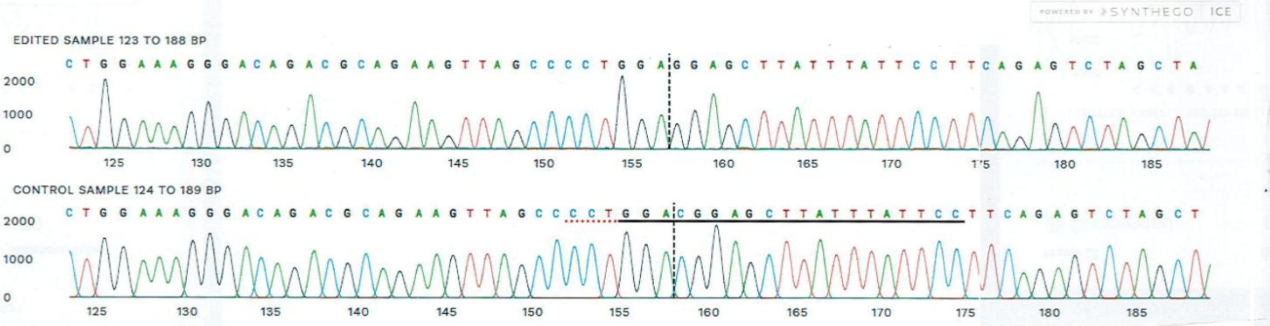

This is the Sanger sequence view showing edited and wild-type (control) sequences in the region around the guide sequence. This shows sequence base calls from both the control and the experimental sample .ab1 files, which will contain mixed base calls. The horizontal black [illegible]

Clone 22A11….. LAP2a +/+

YOUR UPLOAD

LABEL	GUIDE SEQUENCE(S)	CONTROL FILE	EXPERIMENT FILE
**H9C2 22A11 245R 270622_Premixed-TS03543468-copie**	GGAATAAATAAGCTCCGTCC	CTRL2 245 090522_Premixed-TS03340827-copie.ab1	H9C2 22A11 245R 270622_Premixed-TS03543468-copie.ab1

Analysis of H9C2 22A11 245R 270622_Premixed-TS03543468-copie

Contributions | **Indel Distribution** | **Traces**

Status	Guide Target	PAM Sequence	Indel %	Model Fit (R^2)
Succeeded	GGAATAAATAAGCTCCGTCC	AGG	0	1

POWERED BY SYNTHEGO ICE

RELATIVE CONTRIBUTION OF EACH SEQUENCE (NORMALIZED)

	INDEL	CONTRIBUTION	SEQUENCE
+	0	100%	ACTCTGAAGGAATAAATAAGCTCCG ¦ TCCAGGGGCTAACTTCTGCGTCTGTCCCTTTCCAGGCAAGGCTGTATCAG

POWERED BY SYNTHEGO ICE

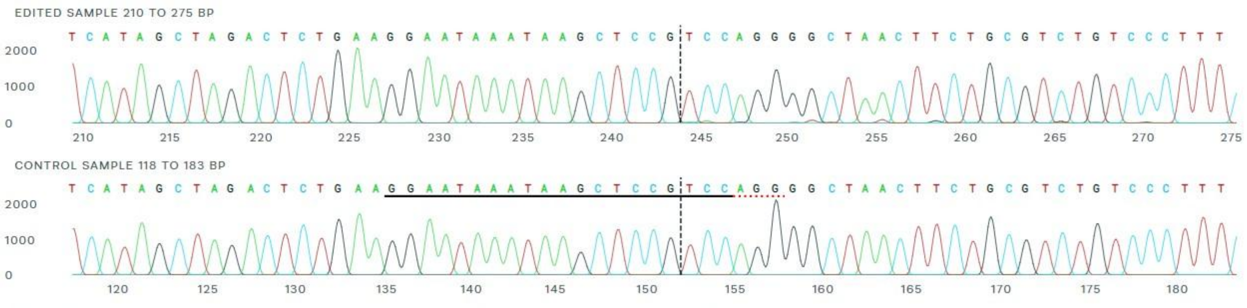

Clone 21E10….. LAP2a +/+

Analysis of 21E10

Contributions | Indel Distribution | Traces

Status	Guide Target	PAM Sequence	Indel %	Model Fit (R^2)
Succeeded	TGAGGCTCCTCAGCACGCGA	TGG	0	1

POWERED BY SYNTHEGO ICE

RELATIVE CONTRIBUTION OF EACH SEQUENCE (NORMALIZED)

	INDEL	CONTRIBUTION	SEQUENCE
+	0	100%	CTGAAACATGAGGCTCCTCAGCACG ¦ CGATGGCTGAGCTCTACTCTTCCCTCCACGGCAAATATTTTCTACTACGT

> The contributions show the inferred sequences present in your edited population and their relative proportions (in contrast to the Indel plot (Indel Distribution tab) that does not specify sequence contributions). Cut sites are represented by black vertical dotted lines, and the wild-type sequence is marked by a "+" symbol on the far left.

Clone 21B1….. LAP2a +/+

Analysis of 21B1 446F 210223_Premixed-TS03935064

Contributions | Indel Distribution | Traces

Status	Guide Target	PAM Sequence	Indel %	Model Fit (R^2)
Succeeded	TGAGGCTCCTCAGCACGCGA	TGG	0	1

POWERED BY SYNTHEGO ICE

RELATIVE CONTRIBUTION OF EACH SEQUENCE (NORMALIZED)

	INDEL	CONTRIBUTION	SEQUENCE
+	0	100%	AGGGAAGAGTAGAGCTCAGCCATCG ¦ CGTGCTGAGGAGCCTCATGTTTCAGATCAGTCAGTTTTCTCTAGTGAAAG

Clone 21H4…… LAP2a +/-

YOUR UPLOAD

LABEL	GUIDE SEQUENCE(S)	CONTROL FILE	EXPERIMENT FILE
**21H4 446R 210622_Premixed-TS03500310-copie**	TGAGGCTCCTCAGCACGCGA	10C4 446R 130622_Premixed-TS03500263-copie.ab1	21H4 446R 210622_Premixed-TS03500310-copie.ab1

Analysis of 21H4 446R 210622_Premixed-TS03500310-copie

Contributions | **Indel Distribution** | **Traces**

Status	Guide Target	PAM Sequence	Indel %	Model Fit (R^2)	Knockout-Score
Succeeded<br>Last guide at position 208 of 287, consider repositioning primers around the cutsites., WARNING - Inf. window after cutsite, 45, is less than 3x indel_max_size of 20, Inference on a short reading window. Possible large deletions or shorter than average readable sequence length.	TGAGGCTCCTCAGCACGCGA	TGG	83	0.99	83

POWERED BY SYNTHEGO ICE

RELATIVE CONTRIBUTION OF EACH SEQUENCE (NORMALIZED)

INDEL	CONTRIBUTION	SEQUENCE
-2	83%	A G G G A A G A G T A G A G C T C A G C C A T C G ¦ - - T G C T G A G G A G C C T C A T G T T T C A G A T C A G T C A G T T T T C T C T A G T G A A A G
+ 0	16%	A G G G A A G A G T A G A G C T C A G C C A T C G ¦ C G T G C T G A G G A G C C T C A T G T T T C A G A T C A G T C A G T T T T C T C T A G T G A A A G

POWERED BY SYNTHEGO ICE

EDITED SAMPLE 172 TO 237 BP

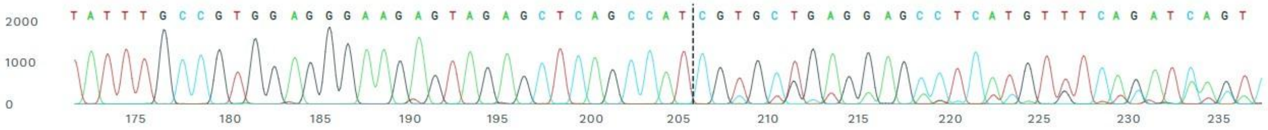

CONTROL SAMPLE 173 TO 238 BP

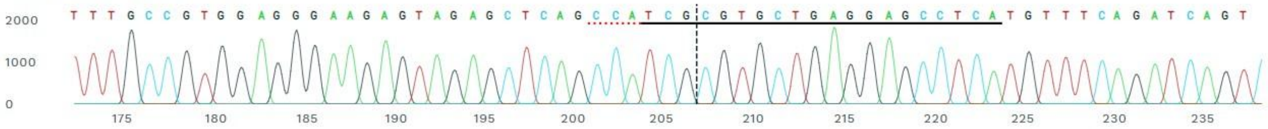

Supplement: Supplementary file 1 — Supplementary data 1. [file ijmsv23p0741s1.pdf]
