# Supplementary material for: LAP2 Isoform Profile in Heart Ageing and in Cardiac Cell Proliferation and Differentiation: Input From CRISPR-Cas9-mediated LAP2a Knockdown in H9C2
Source: Int J Med Sci. 2026 Jan 21;23(3):741–57. doi: 10.7150/ijms.114095 (PMC12964562; doi:10.7150/ijms.114095)
Supplement: Supplementary file 2 — Supplementary data 2. [file ijmsv23p0741s2.pdf]

LAP2 isoform profile in heart ageing and in cardiac cell proliferation and differentiation: input from CRISPR-Cas9-mediated LAP2a knockdown in H9C2

Nathalie Vadrot[1,2], Maryline Moulin[1,3], Ana Ferreiro[1,4,5], Pascale Richard[6,7] and Brigitte Buendia[1,2]

Supplementary Data S2

Lap2a mRNA levels in H9C2 cells :

primers for RT-qPCR were designed upstream of the position of the KO mutations introduced by CRISPR-Cas9

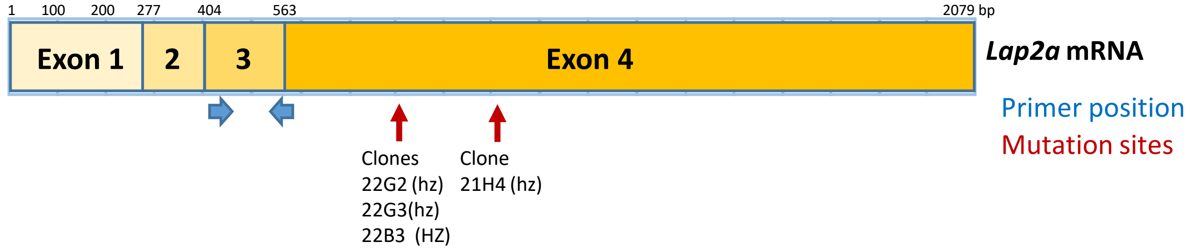

Supplementary Data S2. Schematic diagram of the *Lap2a* mRNA sequence, showing domains corresponding to exons 1 to 4 (NM_001395714.1), and highlighting the position of the primers used for the RT-qPCR experiments (blue arrows), and the position of the mutations triggered by CRISPR-Cas9 for the H9C2 clones of interest (red arrows). Clones with either heterozygote or homozygote mutations are indicated by the abbreviations hz and HZ, respectively.

Rattus norvegicus thymopoietin (Tmpo), transcript variant 1, mRNA
NCBI Reference Sequence: NM_001395714.1
